# Bacterial Lipopolysaccharides Suppress Erythroblastic Islands and Erythropoiesis in the Bone Marrow in an Extrinsic and G- CSF-, IL-1-, and TNF-Independent Manner

**DOI:** 10.3389/fimmu.2020.583550

**Published:** 2020-10-06

**Authors:** Kavita Bisht, Joshua Tay, Rebecca N. Wellburn, Crystal McGirr, Whitney Fleming, Bianca Nowlan, Valerie Barbier, Ingrid G. Winkler, Jean-Pierre Levesque

**Affiliations:** Mater Research Institute – The University of Queensland, Woolloongabba, QLD, Australia

**Keywords:** anemia of inflammation, erythropoiesis, erythroblastic islands, macrophages, lipopolysaccharides, bone marrow, hematopoietic stem cells

## Abstract

Anemia of inflammation (AI) is the second most prevalent anemia after iron deficiency anemia and results in persistent low blood erythrocytes and hemoglobin, fatigue, weakness, and early death. Anemia of inflammation is common in people with chronic inflammation, chronic infections, or sepsis. Although several studies have reported the effect of inflammation on stress erythropoiesis and iron homeostasis, the mechanisms by which inflammation suppresses erythropoiesis in the bone marrow (BM), where differentiation and maturation of erythroid cells from hematopoietic stem cells (HSCs) occurs, have not been extensively studied. Here we show that in a mouse model of acute sepsis, bacterial lipopolysaccharides (LPS) suppress medullary erythroblastic islands (EBIs) and erythropoiesis in a TLR-4- and MyD88-dependent manner with concomitant mobilization of HSCs. LPS suppressive effect on erythropoiesis is indirect as erythroid progenitors and erythroblasts do not express TLR-4 whereas EBI macrophages do. Using cytokine receptor gene knock-out mice LPS-induced mobilization of HSCs is G-CSF-dependent whereas LPS-induced suppression of medullary erythropoiesis does not require G- CSF-, IL- 1-, or TNF-mediated signaling. Therefore suppression of medullary erythropoiesis and mobilization of HSCs in response to LPS are mechanistically distinct. Our findings also suggest that EBI macrophages in the BM may sense innate immune stimuli in response to acute inflammation or infections to rapidly convert to a pro-inflammatory function at the expense of their erythropoietic function.

## Introduction

Anemia of inflammation (AI) is the second most common cause of anemia after iron deficient anemia, and affects 40% of the one billion individuals with anemia worldwide (1). Anemia of inflammation most frequently occurs as a consequence of sepsis (2), infections by bacteria, viruses or fungi, chronic diseases such as inflammatory bowel disease (IBD) (3, 4) and rheumatoid arthritis (5), and hematological malignancies and autoimmune disorders. Anemia of inflammation is multifactorial and involves in part excessive iron sequestration or reduced bioavailability, impaired erythropoietin production and shortened erythrocyte lifespan (1, 6–10). It is assumed that AI is mostly mediated by a combination of these three processes. However, iron and erythropoietin supplementation fail to correct AI in approximately 50% of patients with chronic inflammation such as IBD (3, 4). Furthermore, blood parameters from AI and iron-deficient anemia (IDA) are different. Iron-deficient anemia is characterized by microcytic hypochromatic erythrocytes, low plasma ferritin: soluble transferrin receptor (sTrfR) ratio whereas true AI is normocytic, normochromatic (11) with high plasma ferritin: sTrf ratio (12, 13). Therefore, alternative mechanisms are at work to cause AI.

In the particular case of sepsis, patients are generally not anemic at admission to emergency department but develop anemia once admitted to intensive care units (14). It has been proposed that anemia of sepsis is correlated with (a) intravenous fluid administration during intensive care, which may dilute erythrocytes and hemoglobin of sepsis patients and (b) renal failure (14). In mouse models of acute sepsis involving the systemic administration of bacterial endotoxin such as lipopolysaccharides (LPS), bacterial endotoxins reduce the number of nucleated erythroid cells and reticulocytes and suppress erythropoiesis and iron incorporation in the bone marrow (BM) whilst increasing splenic stress erythropoiesis (15, 16). However the mechanisms by which bacterial endotoxins suppress medullary erythropoiesis have not been extensively delineated.

Erythropoiesis is the crucial process by which hematopoietic stem cells (HSCs) differentiate into erythroid progenitors which mature into enucleated reticulocytes to form blood erythrocytes (Supplementary Figure 1). Adult humans produce ∼2.5 million erythrocytes per second throughout lifespan (17) whereas an adult mouse generates 7,000 erythrocytes per second in steady-state. Erythropoiesis involves differentiation of HSCs into megakaryocyte-erythroid progenitors (MEPs) (18) which further commit to erythroid lineage and generate erythropoietic precursors beginning with erythroid burst-forming units (BFU-E), erythroid colony-forming units (CFU-E), nucleated pro-erythroblasts and followed by basophilic, polychromatic, and orthochromatic erythroblasts (18–20). Erythropoiesis concludes with the enucleation of orthochromatic erythroblasts into immature reticulocytes which matures into erythrocytes with the typical biconcave shape (Supplementary Figure 1). The terminal stage of erythropoiesis occurs within specialized BM niches called erythroblastic islands (EBIs) where maturing erythroblasts rosette and interact with a central ferritin-rich macrophage called erythroblastic island macrophage (EBI Mφ) (21–24). Erythroblastic island macrophage provides growth factors such as insulin-like growth factor-1 (IGF-1) supporting erythroblasts proliferation and differentiation, as well as Fe^2+^ ions for hemoglobin synthesis (25–28). The EBI Mφ is also necessary for erythroblast enucleation into reticulocytes (29–32).

As macrophages are pivotal effectors of innate immunity in response to infections (33), and pivotal regulators of iron homeostasis (27), we hypothesized that EBI Mφ in the BM could play an important role in in the reduction of medullary erythropoiesis associated with bacterial infections, leading to AI. In this study, we demonstrate that LPS, a major component of gram-negative bacteria wall, dramatically suppress medullary erythropoiesis and EBIs. We show the critical role of toll-like receptor-4 (TLR-4) and myeloid differentiation primary response 88 (MyD88) adaptor in suppression of medullary erythropoiesis and EBIs a in response to LPS and provide evidence that this effect of LPS is indirect, possibly via EBI macrophages, and does not require signaling mediated by granulocyte colony-stimulating factor (G-CSF), interleukin (IL)-1, or tumor necrosis factor (TNF).

## Materials and Methods

### Mice and *in vivo* Treatments

C57BL/6 mice were purchased from the Animal Resource Centre (Perth, Australia). B6.129S-*Tnfrsf1a*^TM 1*Imx*^ *Tnfrsf1b*^TM 1*Imx*^/J (*Tnfrsf1a^–/–^; Tnfrsf1b^–/–^*), B6.129S7-*Il1r1*^TM 1*Imx*^/J (*Il1r1^–/–^*) and B6.129 × 1(Cg)-*Csf3r*^TM 1*Link*^/J (*Csf3r*^–/^*^–^*) mice backcrossed more than 10 times in C57BL/6 background were purchased from Jackson Laboratory. *Myd88*^TM 1*Aki*^ (*Myd88^–/–^*) (34) and *Tlr4*^TM 1*Aki*^ (*Tlr4*^–/–^) (35) mice were donated by Dr. Antje Blumenthal (The University of Queensland Diamantina Institute). All mice had been backcrossed more than 10 times in C57BL/6 background and were maintained at The University of Queensland Biological Resources Facility at the Translational Research Institute. All mice used were males and aged between 8 and 12 weeks. Procedures were approved by The University of Queensland Health Sciences Animal Ethics Committee (105/15, 327/16, and 313/19). γ-irradiated purified LPS from *Escherichia coli* strain 0111:B4 (Sigma-Aldrich catalog # L4391) was administered at 2.5 mg/kg body weight intraperitoneally daily for two consecutive days; control mice received an equivalent volume of saline. Bone marrow tissue, blood, and spleens were harvested at specified time-points for subsequent analyses.

### Tissue Harvest

At the endpoint of the experiments, mice were anesthetized with isoflurane and 0.5–1 mL of blood collected into heparinized tubes by cardiac puncture before cervical dislocation. The BM of one femur was flushed into 1 mL phosphate buffered saline (PBS) containing 2% fetal calf serum (FCS). Blood was centrifuged twice at 800G for 10 min and plasma collected, aliquoted and stored at −80°C. Spleens were dissociated in 3 mL PBS 2% FCS using a GentleMACS Dissociator tissue homogenizer with matching C tubes (Miltenyi Biotec, Macquarie Park, Australia) on “spleen 3” setting, twice. For flow cytometry analyses, red cells were lysed from blood samples as previously described (36).

Blood was diluted 1:2 in PBS and counted on Mindray BC-5000 Vet Auto hematology analyzer (Biomedical Electronics Co. LTD., China). Spleen and BM samples were counted on Coulter Counter (Beckman Coulter).

### In-Flight Imaging Flow Cytometry

Extraction and staining of BM aggregates was performed as described previously (37, 38). In brief, harvested femurs were gently flushed multiple times with a 25G needle and 1 mL syringe containing ice cold IMDM supplemented with 20% FBS, 100 U/mL penicillin, 100 μg/mL streptomycin, 2 mM L-glutamine (all from Thermo Fisher Scientific, Waltham, MA) to dislodge most of the BM cells. Bone marrow suspension containing 10^7^ leukocytes were fixed with 4% paraformaldehyde in PBS for 10 min at room temperature. Cell suspensions were washed twice in PBS containing 2% newborn calf serum (NCS) and stained with Hoechst33342 (Hoe), CD169-FITC, CD71-PE, CD11b-PECF594, anti-Ter119-PercPCy5.5, anti-VCAM-1-PECy7, anti-F4/80-APC and anti-LY6G-APCCy7 antibodies (Supplementary Table 1). Samples were acquired within 6 h of preparation, using INSPIRE software on an Amnis ImageStream^*X*^ Mk II equipped with 405, 488, and 642 nm lasers (Luminex, Austin, TX) and detected and quantified using IDEAS 6.2 software (Luminex) as previously described (38). The number of EBIs per 10 million BM cells (*N_10__*mil*_*) was calculated by the following formula: $N_{10\,m\,i\,l}=\frac{N_{r\,a\,w}\times C_{f\,e\,m\,u\,r}}{P_{f\,e\,m\,u\,r}\times P_{a\,n\,a\,l\,y\,z\,e\,d}\times 10^{7}\,c\,e\,l\,l\,s}$, where *N*_*raw*_ = number of EBIs counted on IDEAS software, *C*_*femur*_ = number of BM cells per femur, *P*_*femur*_ = proportion of femur (by volume) stained with antibodies, and *P*_*analyzed*_ = volumetric proportion of BM sample run.

### Conventional Flow Cytometry

All the antibodies used in this study were from BioLegend except for anti-Flk-2/Flt3 antibody (BD Bioscience). Antibody conjugates, clones and dilutions are specified in Supplementary Table 1. Blood or spleen samples were stained in suspension on ice for 40 min in mouse CD16/CD32 hybridoma 2.4G2 supernatant containing fluorescein isothiocyanate (FITC)-conjugated lineage antibody cocktail (CD3ε, CD5, anti-B220, CD11b, anti-Ter119, and anti-Gr-1) together with anti-KIT-allophycocyanin (APC), CD150-phycoerythrin (PE), anti-Sca-1-PE-Cyanin7 (PECy7), CD48-Pacific blue (PB), CD45-APC-Cyanin7 (APCCy7), and anti-Flk2/Flt3-PE-CF594 to measure HSPC mobilization. To identify pro-erythroblasts, maturing erythroblasts and enucleated erythroid cells, BM or spleen samples were stained with antibody cocktail containing anti-Ter119-FITC, Hoechst33342 (Hoe; Sigma-Aldrich), CD44-APC and CD45-APCCy7 antibodies. 7-actinomycin D (AAD; Invitrogen) was added to all stained samples for dead cell exclusion. To stain myeloid cells, BM was stained with biotinylated lineage antibody cocktail (CD3ε, anti-B220, CD49b) with streptavidin- brilliant ultraviolet 395 (BUV395), CD11b-brilliant violet 510 (BV510), anti-F4/80-APC, anti-VCAM-1-PECy7, CD169-PE/FITC, anti-Ly6G-FITC/APCy7, and CD45-BV785 antibodies. Samples were analyzed either on a CyAn 9C (Beckman Coulter) or a Cytoflex (Beckman Coulter) flow cytometer equipped with 640, 561, 488, and 405 nm lasers. Uncompensated FCS files were analyzed using FlowJo10 software (Tree Star, Ashland, OR following compensation with single color antibody stains) following *post hoc* compensation with single color controls.

To analyze TLR-4 expression by flow cytometry, anti-TLR-4-PE antibody was added to myeloid, erythroblasts and erythroid progenitor antibody cocktail and TLR-4 expression was analyzed on a BD LSR Fortessa X20 (BD Biosciences, San Jose, CA) flow cytometer equipped with 3 or Fortessa (BD Bioscience) flow cytometer equipped with 633nm, 561nm, 488nm, 405nm and 355nm lasers. The erythroid progenitor antibody cocktail was made of biotinylated lineage antibody cocktail (CD3ε, CD5, anti-B220, CD11b, anti-Ter119, and anti-Gr-1) with streptavidin- brilliant ultraviolet 395 (BUV395), anti-KIT-APCCy7, anti-Sca1-PECy5, CD150-PECy7, CD48-PB, CD41-FITC, CD16/32-PercpCY5.5 and CD105-APC, and anti-TLR-4-PE.

### Cell Sorting and Quantitative Real-Time Reversed Transcribed Polymerase Chain Reaction

Fluorescence-activated cell sorting of BM erythroid progenitors, erythroid and myeloid cells and quantitative real-time reversed transcribed polymerase chain reaction (qRT-PCR) analyses were performed as previously described (19, 39, 40). Briefly, mouse femurs were flushed with 1 mL PBS containing 2% FCS and 2 mM EDTA, and cells were stained with myeloid antibody cocktail as described above. Ter-119^–^ CD11b^+^ F4/80^+^ Ly6G^–^ CD169^+^ VCAM-1^+^ macrophages (referred to CD11b^+^ Mφ), Ter-119-CD11b^+^ F4/80^+^ Ly6G-CD169-VCAM-1-Ly6C bright (monocytes) and Ter119-CD11b+F4/80-Ly6G+ (neutrophil) were directly sorted into Trizol LS (ThermoFisher Scientific, Waltham, MA) using BD FACSAria Fusion sorter (BD Bioscience, San Jose, CA). To sort erythroblasts, BM was stained with anti-Ter119-FITC, anti-CD44-APC, and anti-CD45-BV785 and cells were sorted into Trizol LS. To sort erythroid progenitors, BM was stained with brilliant ultraviolet395 (Buv395) conjugated lineage antibody cocktail (CD3ε, CD5, anti-B220, CD11b, anti-Ter119 and anti-Gr-1) together with anti-KIT-APCCy7, anti-CD48-PB, anti-CD150-PECy7, anti-CD41-FITC, anti-CD34-PE, anti-CD105-APC anti-CD16/32-PerCPCy5.5 and CD-45 BV785. Pre-MegE (Lin^–^ Sca^–^ KIT^+^ CD16/32^–^ CD150^+^ CD105^–^), pre-CFU-E (Lin^–^ Sca^–^ KIT^+^ CD16/32^–^CD150^+^ CD105^+^) and CFU-E (Lin^–^ Sca^–^ KIT^+^ CD16/32^–^ CD150^–^ CD105^+^) cells as described previously (39, 41) were sorted into Trizol LS. Dead cells were excluded using fixable viability stain (FVS)700 (BD Horizon). RNA was then extracted using GeneJet RNA Clean up and Concentration Micro Kit (ThermoFisher Scientific) and cDNA was synthesized with SensiFast cDNA synthesis kit (Bioline, Alexandria NSW, Australia). Quantitative real-time reversed transcribed polymerase chain reaction was performed with TaqMan Fast Advanced Mix (ThermoFisher Scientific) for mouse *Hprt* (Mm03024075_m1), *Tlr4* (Mm00445274_m1), primer/probe sets (ThermoFisher Scientific) using ViiA 7 Real-for Time PCR system (ThermoFisher Scientific).

### Colony Forming Assays

Ten microliter whole blood or splenocytes from 1/1000 of a whole spleen were seeded in 35 mm petri dishes and covered with 1 mL Iscove’s modified Dulbecco’s medium (IMDM) supplemented with 1.6% methylcellulose, 20% FCS and optimal concentrations of conditioned media containing recombinant mouse IL-3, IL-6, and KIT ligand as previously described (42).

### BFU-E Colony Assay

Erythroid burst-forming units (BFU-E) colony assay was performed as previously described (41) with minor modifications. 50,000/mL BM cells were plated in MethoCult^TM^ SF M3436 medium (Stem Cell Technologies) containing 6 μg/mL Fluconazole (Diflucan), 50 ng/mL recombinant mouse KIT ligand (Biolegend), 4 international units/mL recombinant human erythropoietin (Jensen) and BIT9500 serum substitute (Stem Cell Technologies). In some dishes, 100 ng/mL of LPS was added into the medium. BFU-E colonies were counted on day 14 of cultured at 37°C in the presence of 5% CO_2_.

### Enzyme Linked ELISA

Mouse G-CSF was quantified in mouse plasma using commercially available ELISA kit diluted according to manufacturer’s instructions (R&D Systems).

### Statistics

Data are presented as mean ± SD. Statistical differences were calculated using two-sided Mann-Whitney test between two groups, and one-way ANOVA with Tukey’s *post hoc* test or Kruskal-Wallis test with Dunn’s *post hoc* test for multiple comparisons using GraphPad Prism 7.03 (La Jolla, CA).

## Results

### LPS Suppresses Medullary Erythropoiesis

**FIGURE 1:**
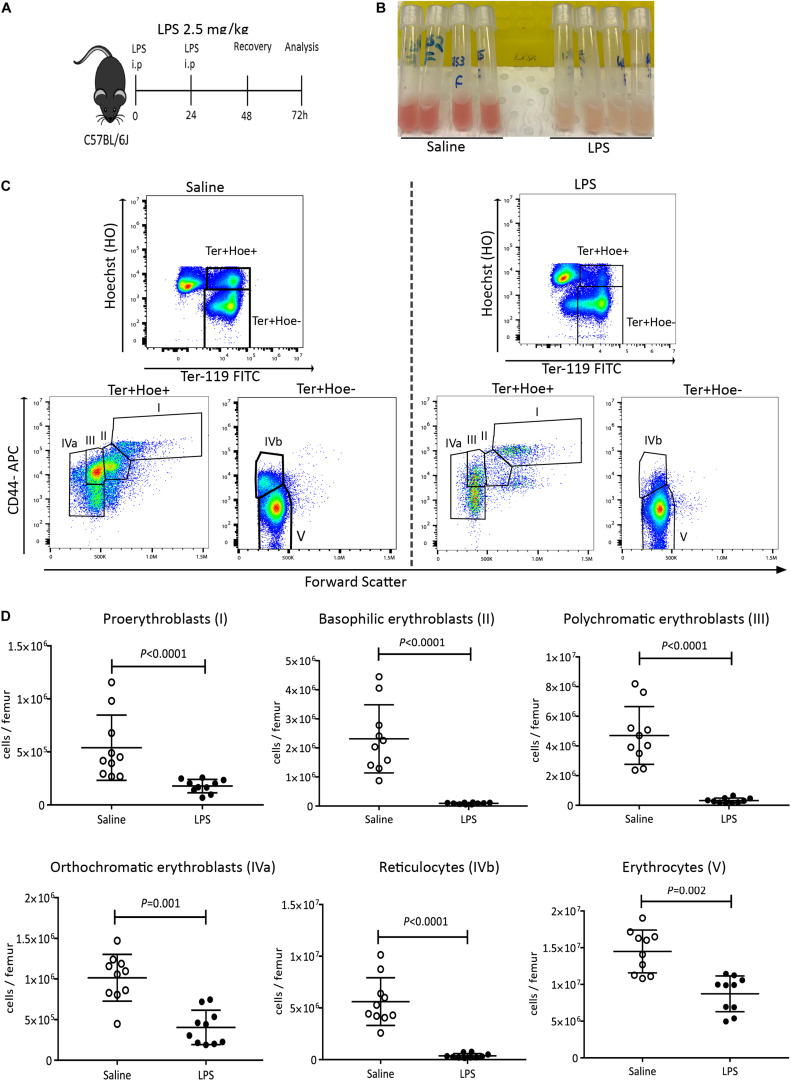
LPS administration suppresses medullary erythropoiesis. **(A)** C57BL/6 mice were administered saline or LPS for 48 h and harvested after 2 days of recovery. **(B)** Photographs of femoral BM flushed from separate mice into 1 mL PBS after saline or LPS treatment. Note the discoloration of BM in mice treated with LPS. **(C)** Representative flow cytometry plots showing the changes in erythroblasts and reticulocytes after LPS or saline treatment. Nucleated pro-erythroblasts and erythroblasts were gated as Hoe^+^ Ter119^+^ cells and segregated as pro-erythroblasts (I), basophilic erythroblasts (II), polychromatic erythroblasts (III) and orthochromatic erythroblasts (IVa) according to CD44 expression and forward scatter (cell size). Enucleated reticulocytes (IVb) and erythrocytes (V) were gated from the Hoe^–^ Ter119^+^ population. **(D)** Quantification of proerythroblasts, erythroblasts, reticulocytes and erythrocyte populations in the femoral BM. Data are from 2 pooled experiments performed at several months interval. Each dot represents an individual mouse. *n* = 7–8 mice per group. Bars are means ± SD. Statistical significance was determined using two-tailed Mann Whitney test.

To test the effect of LPS on medullary erythropoiesis, C57BL/6J mice were injected with LPS at 2.5 mg/kg daily or saline for two consecutive days and femoral BM cells was flushed in 1mL PBS at 48 h post LPS treatment (Figure 1A). We noticed a marked discoloration of the red BM 48 h after LPS treatment (Figure 1B) suggesting suppressed medullary erythropoiesis. BM cells were stained with Hoechst33342 (Hoe), Ter119, and CD44 antibodies to quantify by flow cytometry the number of nucleated Hoe^+^ pro-erythroblasts (population I), basophilic erythroblasts (II), polychromatic erythroblasts (III), orthochromatic erythroblasts (IVa) and enucleated Hoe^–^ reticulocytes (IVb) and erythrocytes (V) (Figure 1C) following a previously published strategy (7, 19) in which we added Hoe in the staining cocktail to better resolve nucleated orthochromatic erythroblasts from enucleated reticulocytes in populations IVa and IVb. This confirmed profound reductions in the numbers of basophilic and polychromatic erythroblasts and reticulocytes, with a 50–60% reduction in the numbers of pro-erythroblasts, orthochromatic erythroblasts and erythrocytes in the BM (Figures 1C,D). These erythroid populations were still significantly reduced 5 days post-LPS treatment despite some recovery (Supplementary Figures 2A–D). Whole blood counts at 48 h after LPS administration showed dramatic increases in neutrophils, monocytes, eosinophils, basophils (Supplementary Figures 3B–E) and significant reduction in lymphocytes and platelets (Supplementary Figures 3F,G). Of note, due to the 40-day half-life of mouse erythrocytes, erythrocytes numbers and volumes, hematocrit and hemoglobin concentrations were not affected at this early time-point after LPS challenge (Supplementary Figures 3H–L) consistent with the lack of anemia in the early stage of sepsis in human patients (14).

As it is known that extramedullary erythropoiesis takes place in the mouse spleen after infection or stress (15, 43–47), we confirmed increased number of proerythroblasts and erythroblasts in the spleen 48 h following LPS treatment (Supplementary Figures 4A–D). Since the mechanisms of medullary erythropoiesis suppression in response to LPS have not been extensively studied, we focused on erythropoiesis in the BM in subsequent experiments.

### LPS Suppresses Medullary Erythropoiesis via TLR-4 and MyD88

**FIGURE 2:**
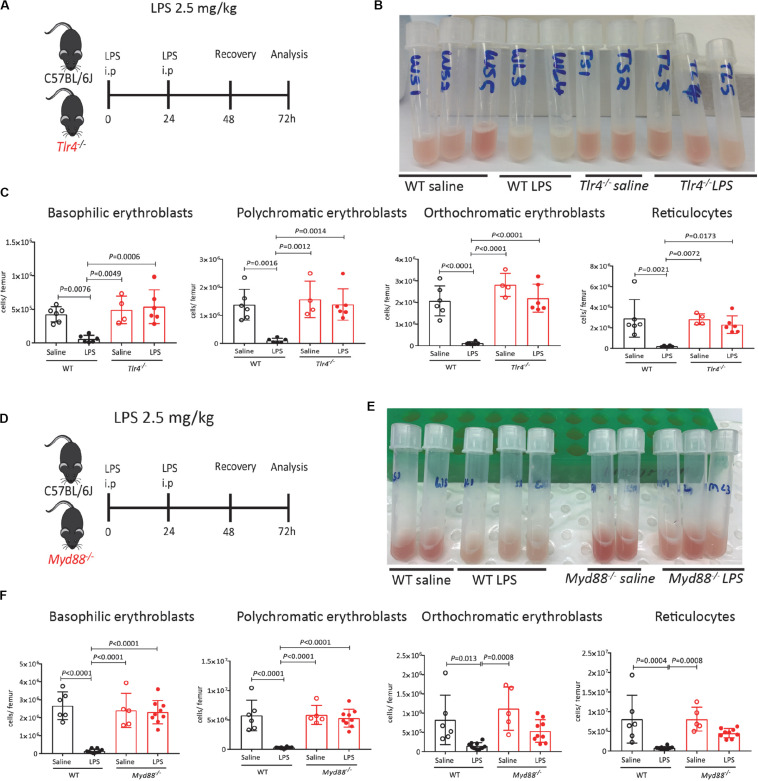
Suppression of medullary erythropoiesis by LPS is mediated by TLR-4 and MyD88. **(A)** C57BL/6 (WT) and *Tlr4*^–^*^/^*^–^ mice were administered saline or LPS and harvested after 48 h of recovery. **(B)** Photographs of mouse femoral BM flushed into 1 mL PBS after LPS or saline treatment. Note the redness of BM in *Tlr4*^–^*^/^*^–^ mice treated with LPS. **(C)** Quantification of erythroblasts and reticulocytes in the femoral BM. **(D)** C57BL/6 (WT) and *Myd88*^–^*^/^*^–^ mice were administered saline or LPS and harvested after 48 h of recovery. At harvest, the number of proerythroblasts, erythroblasts and reticulocytes were measured in the BM. **(E)** Photographs of mouse femoral BM flushed into 1 mL PBS after LPS or saline treatment. Note the redness of BM in *Myd88*^–^*^/^*^–^ mice treated with LPS. **(F)** Quantification of erythroblasts and reticulocytes in the femoral BM. Data are from 2 pooled experiments performed at several months interval. Each dot represents an individual mouse. *n* = 4–6 mice per group. Bars are means ± SD. Statistical significance was determined using one-way ANOVA with Tukey’s multiple comparison test.

Lipopolysaccharides activate innate immune cells primarily via its canonical receptor TLR-4. We therefore first tested the effects of LPS on HSPC mobilization and medullary erythropoiesis in mice defective for the TLR-4 gene (*Tlr4*^–/–^). Wild-type C57BL/6 mice robustly mobilized colony-forming cells (CFCs), Lin^–^ Kit^+^ Sca1^+^ hematopoietic stem and progenitor cells (HSPCs) and phenotypic Lin^–^ Kit^+^ Sca1^+^ CD48^–^ CD150^+^ HSCs into the blood and spleen 48 h after LPS challenge. In the absence of functional *Tlr4* gene, HSPCs did not mobilize in response to LPS (Supplementary Figures 5A–F) in agreement with previous reports (48–50). Noticeably, the BM from *Tlr4*^–/–^ mice were not whitened after LPS treatment unlike their WT counterparts (Figures 2A,B). Flow cytometry data confirmed that LPS treatment did not suppress erythroblast and reticulocyte populations in *Tlr4*^–/–^ mouse BM establishing that suppression of medullary erythropoiesis by LPS is also mediated by TLR-4 (Figure 2C).

Myeloid differentiation primary response 88 is a crucial adapter protein for cell surface TLR signaling (all TLRs except TLR-3 and endosomal TLR-4) (51). Previous work has shown that *Salmonella* infection enhances extra-medullary stress erythropoiesis in spleen via MyD88 (52) and reduces zymosan induced erythrophagocytosis in BM-derived monocytes (47). We therefore tested the effect of LPS on medullary erythropoiesis in mice lacking MyD88. Similar to *Tlr4*^–^*^/^*^–^ mice, the BM was not whitened in LPS-treated *Myd88*^–^*^/^*^–^ mice (Figure 2E) and flow cytometry analysis confirmed that LPS required MyD88 to inhibit medullary erythropoiesis (Figure 2F).

### LPS Suppresses Medullary Erythropoiesis Extrinsically

**FIGURE 3:**
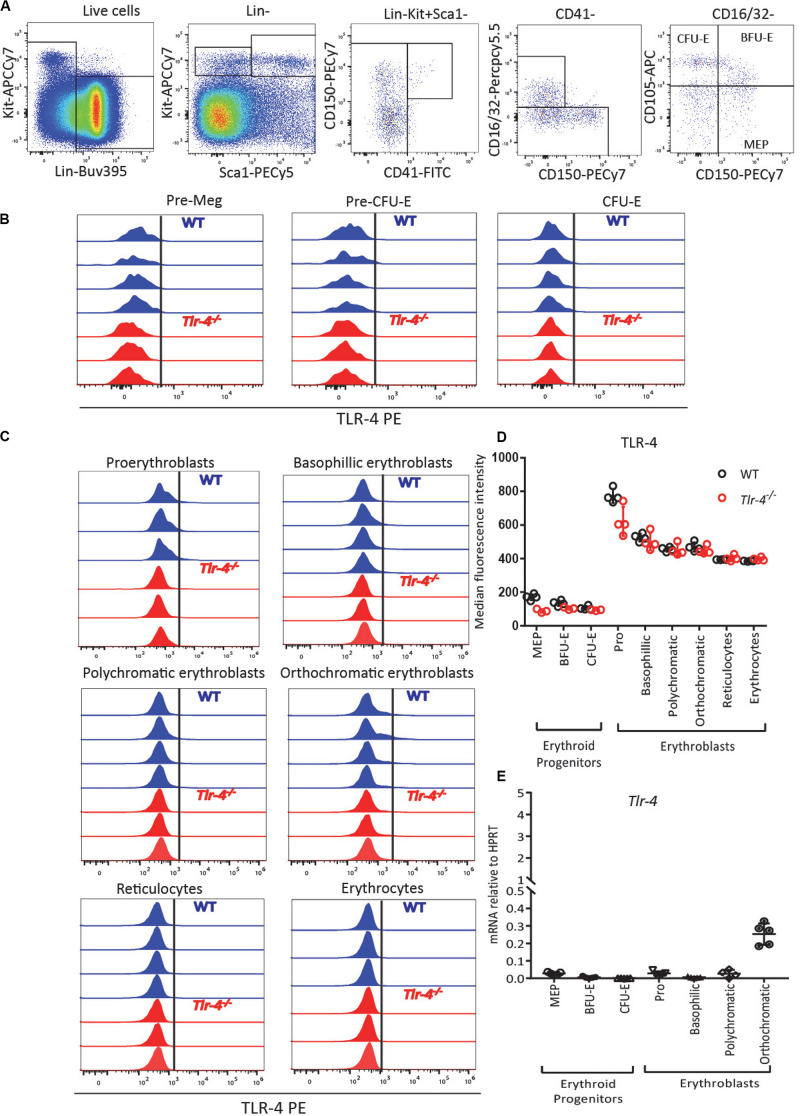
TLR-4 is not expressed by BM erythroid progenitors and erythroblasts. **(A)** Gating strategy for TLR-4 expression on erythroid progenitors. MEPs (Lin^–^ SCA1^–^ KIT^+^ CD16/32^–^ CD150^+^ CD105^–^), BFU-E (Lin^–^ SCA1^–^ KIT^+^ CD16/32^–^CD150^+^ CD105^+^) and CFU-E (Lin^–^ SCA1^–^ KIT^+^ CD16/32^–^ CD150^–^ CD105^+^) from untreated C57BL/6 mice. **(B,C)** Flow cytometry histograms showing TLR-4 is not expressed by erythroid progenitors **(B)** or by pro-erythrobasts, erythroblasts, reticulocytes and erythrocytes **(C)**. Each histogram represents TLR-4 expression profile from a separate WT (blue) or *Tlr4*^–/–^ (red) mouse. **(D)** Median fluorescence intensity of anti-TLR-4 antibody binding showed no difference between WT and *Tlr4*^–^*^/^*^–^ mice. **(E)** qRT-PCR showed for *Tlr4* mRNA relative to *Hprt* in sorted erythroid progenitors and erythroblast populations. In panels **(D,E)**, each dot represents a separate mouse/separate sort (*n* = 3–5 mice per cell population). Bars are means ± SD.

To determine whether LPS mediates its effects directly on erythroblasts or indirectly, we performed flow cytometry for cell surface TLR-4 and qRT-PCR for *Tlr4* gene expression on sorted phenotypic erythroid progenitors (MEPs, BFU-E, and CFU-E) from the BM of untreated C57BL/6 mice using previously published gating strategies (39, 41) (Supplementary Figure 1 and Figure 3A), as well as erythroblast populations as defined in Figure 1C. Flow cytometry performed in WT and *Tlr4*^–^*^/^*^–^ mice as negative controls confirmed that TLR-4 protein is not expressed at the surface of erythroid progenitors, erythroblasts or erythrocytes as there was no difference in anti-TLR-4 antibody binding on cells from WT or *Tlr4*^–/–^ mice (Figures 3B–D). qRT-PCR on sorted cells from WT mice confirmed that *Tlr4* mRNA was not expressed by phenotypic erythroid progenitors or erythroblasts with low levels only detected in orthochromatic erythroblasts suggesting that the effect of LPS is not directly via pro-erythroblasts, polychromatic and polychromatic erythroblasts (Figure 3E). Our results are consistent with the Gene Expression Commons dataset^1^ (53) and the Haemopedia dataset^2^ (54) which both show by gene expression microarray and RNA sequencing on sorted mouse BM cells respectively that *Tlr4* mRNA is not expressed on any erythroid progenitor subset from MEPs to reticulocytes (Supplementary Figure 6).

**FIGURE 4:**
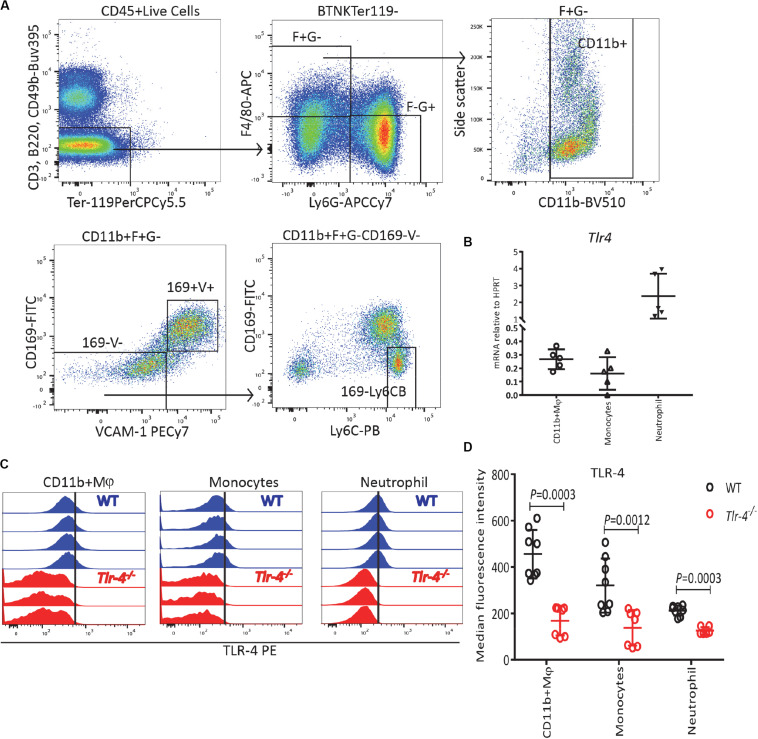
TLR-4 is expressed by BM myeloid cells. **(A)** Gating strategy for TLR-4 expression on myeloid cells: Ter119^–^ CD11b^+^ F4/80^+^ Ly6G^–^ CD169^+^ VCAM-1^+^ macrophages (Mφ), Ter119^–^CD11b^+^ F4/80^+^ Ly6G^–^ CD169^–^ VCAM-1^–^ Ly6C^*bright*^ monocytes and Ter119^–^ CD11b^+^ F4/80^–^ Ly6G^+^ neutrophils. **(B)** qRT-PCR showing *Tlr4* mRNA is expressed by myeloid cells. **(C)** Flow cytometry histograms for TLR-4 expression in WT and *Tlr4*^–^*^/^*^–^ on myeloid cells. Each histogram represents TLR-4 expression profile from a separate WT (blue) or *Tlr4*^–/–^ (red) mouse. **(D)** Median fluorescence intensity for TLR-4 on myeloid cells in WT mice (blue) compared to *Tlr4*^–^*^/^*^–^ (red) mice. Data are from 2 pooled experiments performed at several months interval Each dot represents an individual mouse. *n* = 5–8 mice per group. Bars are means ± SD. Statistical significance was determined using two-tailed Mann Whitney test.

Considering that the final stages of erythropoiesis occur in EBIs which contain a central Mφ (28, 31, 32, 55) and that Mφ are known responders to LPS (33), we also analyzed *Tlr4* mRNA expression on sorted BM myeloid cells gated as described in Figure 4A following exclusion of erythroid cells by gating on Ter119^–^ cells. All BM myeloid cells expressed *Tlr4* mRNA including Ter119^–^CD11b^+^F4/80^+^VCAM-1^+^CD169^+^Ly6G^–^ Mφ, Ter119^–^CD11b^+^ F4/80^+^VCAM-1^–^CD169^–^Ly6G^–^ monocytes and particularly Ter119^–^ CD11b^+^F4/80^–^Ly6G^+^ neutrophils (Figure 4B). Flow cytometry performed in parallel on WT and *Tlr4*^–/–^ mice with the same monoclonal anti-TLR-4 antibody further confirmed that TLR-4 is expressed at the surface of all BM myeloid cells including CD11b^+^ Mφ, monocytes and neutrophils (Figures 4C,D). Again, our results are consistent with the Haemopoedia and Gene Expression Commons datasets (Supplementary Figure 6). Together these results suggest that LPS does not inhibit erythropoiesis directly via erythroid progenitors and erythroblasts but indirectly possibly via myeloid cells that express TLR-4.

**FIGURE 5:**
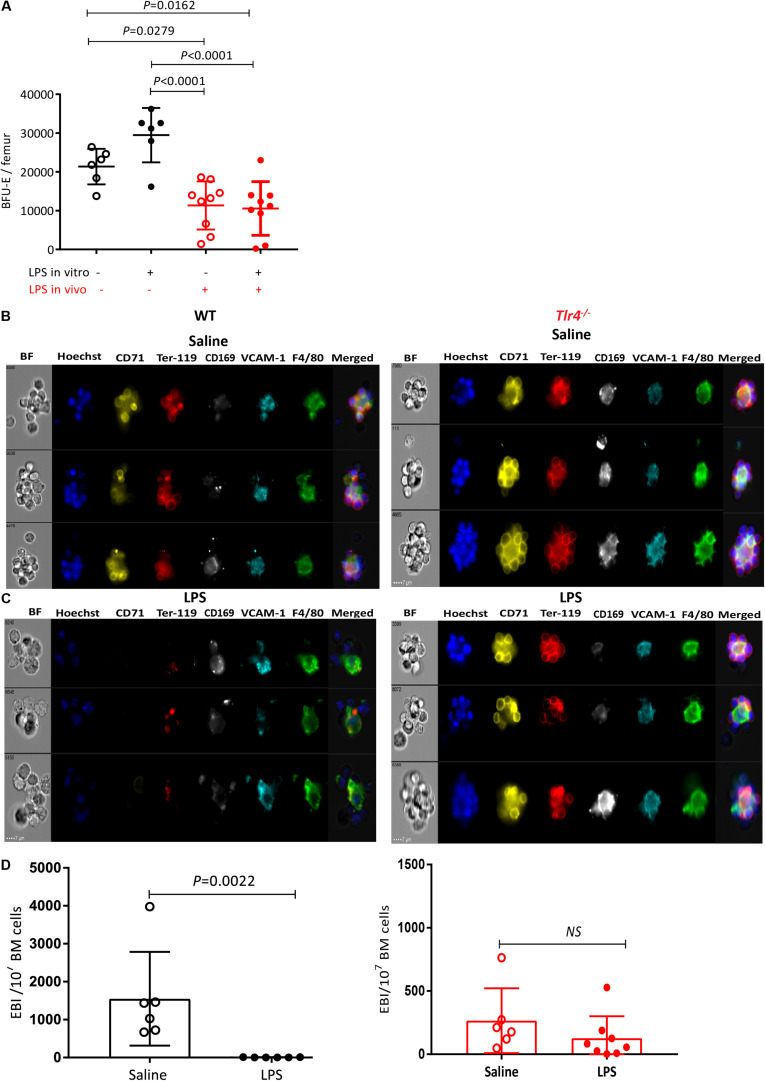
LPS treatment sharply decreases EBI frequency in the BM in a TLR-4-dependent manner. **(A)** BM cells from saline or LPS-treated mice (LPS *in vivo* −/+) were plated in a BFU-E colony assay in the presence or absence of 100 ng/mL LPS in the colony assay medium (LPS *in vitro*±). After 14 days of cultures BFU-E colonies were counted and number of BFU-E per femur was calculated. Each dot represents a separate mouse (each mouse is average of duplicate plates). Bars represent means ± SD. One-way ANOVA with Tukey’s multi comparison test. **(B,C)** Representative photomicrographs of gated EBI (Hoechst33342 (HO)^+^ CD71^+^ Ter119^+^ F4/80^+^ VCAM1^+^ cell aggregates from the BM of mice treated with **(B)** saline or **(C)** LPS. Note that typical EBIs in saline treated mice **(B)** are disrupted after LPS treatment **(C)**. **(D)** Quantification of EBI frequency in the BM of mice treated with saline or LPS. Each dot represents a separate mouse and bars represent means ± SD. Statistical significance was determined using two-tailed Mann Whitney test.

To confirm that LPS suppressive effects on medullary erythropoiesis is indirectly mediated, we first performed BFU-E colony assays. Bone marrow cells were harvested from C57BL/6 mice treated with either saline or LPS *in vivo* for 2 days and then plated in with BFU-E semi-solid medium. In some dishes, 100 ng/mL of LPS was added into the colony assays (*in vitro* treatment). Addition of LPS *in vitro* did not alter erythroid colony numbers (Figure 5A) confirming that the suppressive effect of LPS on medullar erythropoiesis *in vivo* is not a direct effect on erythroid progenitors. However, erythroid colony numbers from the BM of LPS-treated mice (*in vivo* treatment) were significantly reduced by 50% compared to saline treated mice, regardless of LPS presence *in vitro* (Figure 5A) in agreement with previous observations (56). Thus our data suggest that the reduction of BFU-E numbers in the BM of LPS treated mice *in vivo* is indirect.

### LPS Treatment Reduces Frequency of Medullary EBIs

As pro-erythroblast and erythroblasts mature in EBIs containing a central Mφ, we next evaluated the effect of LPS on EBIs in the BM by in-flight imaging flow cytometry to directly visualize and quantify EBIs and their central Mφ (38). In saline-treated mouse BM, we could detect typical EBIs with at least 5 Ter119^+^CD71^+^ erythroblasts and reticulocytes rosetted around a central F4/80^+^ VCAM-1^+^ CD169^+^ EBI Mφ (Figures 5B–D) as previously reported (28, 37, 38). Remarkably, EBIs were disrupted in the BM 48 h after two successive daily LPS injections as suggested by the very low frequency of cell aggregates containing Ter119^+^CD71^+^ erythroblasts rosetted around a central Mφ (Figures 5B–D). In fact, in the BM of LPS-treated mice, most cell aggregates containing a central Mφ were either negative for Ter119 and CD71 or contained Ter119^+^ cell debris (Figure 5C). Furthermore these Mφ had more circular and less reticulated morphology compared to EBI Mφ from the BM of saline-treated mice possibly reflecting a change in macrophage function or a loosening of the adhesive interactions between EBI Mφ and erythroblasts.

In order to determine whether this dramatic reduction in medullary EBI frequency could be an indirect consequence of erythroblast number reduction in the BM (which then could not cluster with EBI Mφ) rather than alteration of EBI Mφ function, we repeated this experiment at earlier time-points during the two day course of LPS administration (Supplementary Figures 7A,B). Twenty-four hours after the first LPS injection, we noted an approximately 60% reduction in the frequency of EBIs which still displayed their typical morphology with over 5 Ter119^+^ CD71^+^ erythroblasts rosetted around a central F4/80^+^ CD169^+^ VCAM-1^+^ Mφ (Supplementary Figures 7D–G). At this same timepoint proerythroblasts were not significantly reduced whereas each erythroblast subset was reduced in similar proportion to EBIs (Supplementary Figure 7C). Twenty-four hours after the second LPS injection (48 h time-point), properly formed EBIs were very rare. Most Ter119^+^ cell aggregates with a central Mφ contained Ter119^+^ cell debris similar to the 48 h after the last LPS injection (Figure 5C). Importantly, the number of proerythroblasts was decreased by only 50% 24 h after the second LPS injection (not significant by one-way ANOVA) whereas all erythroblast subsets were suppressed similar to EBI frequency (Supplementary Figure 7C). These kinetics show that medullary EBIs and erythroblasts decrease very dramatically and in parallel within the first 48 h of LPS administration while proerythroblasts and BFU-E decrease at much slower rates with approximately 50% of proerythroblasts and BFU-E still present 24 and 48 h after the second LPS injection (Figures 1D, 5A and Supplementary Figure 7C). Therefore, the loss of medullary EBIs and erythroblasts in response to LPS is not due to exhaustion of upstream proerythroblasts (Supplementary Figure 7C) or even BFU-E (Figure 5A) which are still present in the BM 24 and 48 h after the last LPS injection.

We repeated this experiment in *Tlr4*^–/–^ mice, and unlike WT mice, typical EBIs with a reticulated central Mφ were clearly detected in the BM of *Tlr4*^–/–^ mice and LPS treatment had no significant impact on the frequency of EBIs in the BM (Figure 5D) showing this effect is TLR-4-dependent and was not mediated by contaminants in the LPS preparation in a TLR4-independent manner.

### LPS Suppresses Medullary Erythropoiesis Independently of IL-1, TNF or G-CSF

**FIGURE 6:**
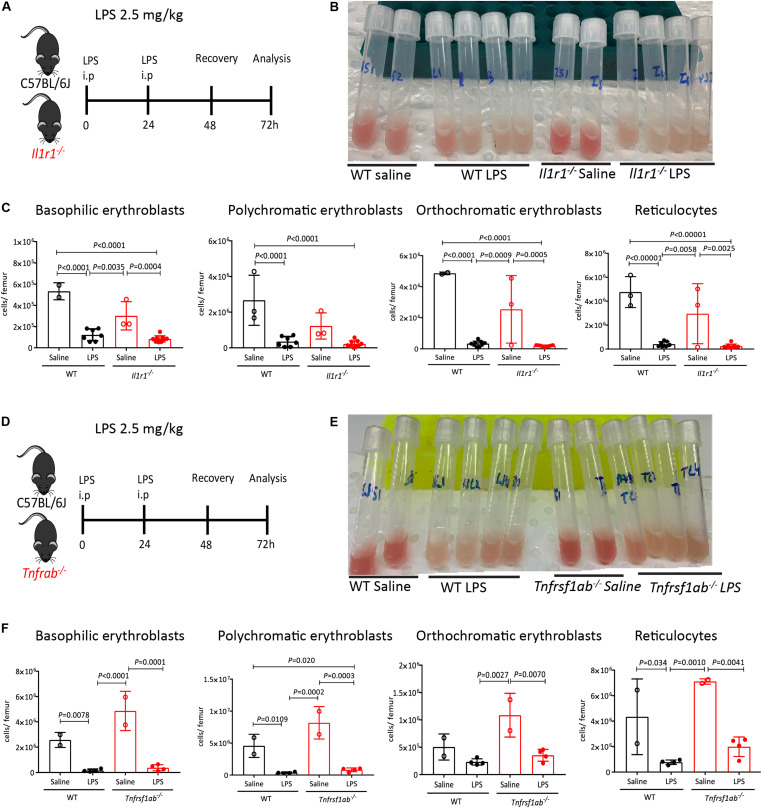
IL-1 receptor and TNF receptors are dispensable for medullary erythropoiesis suppression in response to LPS. **(A)** Experimental design with WT and *Il1r1*^–^*^/^*^–^ mice. **(B)** Photographs of mouse femoral BM flushed into 1 mL PBS after LPS or saline treatment. Note the discoloration of BM in mice treated with LPS. **(C)** Quantification of erythroblasts and reticulocytes populations in the femoral BM of WT and *Il1r1*^–^*^/^*^–^ mice. **(D)** Experimental design with WT and *Tnfrsf1a*^–^*^/^*^–^*; Tnfrsf1b*^–^*^/^*^–^ mice. **(E)** Photographs of mouse femoral BM flushed into 1 mL PBS after LPS or saline treatment. Note the discoloration of BM in mice treated with LPS. **(F)** Quantification of erythroblasts and reticulocytes populations in the femoral BM from WT and *Tnfrsf1a*^–^*^/^*^–^*; Tnfrsf1b*^–^*^/^*^–^ mice. Data are from 2 pooled experiments performed at several weeks interval. *n* = 2–9 per group. Bars are means ± SD Statistical significance was determined using one-way ANOVA with Tukey’s multiple comparison test.

Anemia of inflammation is characterized by the overproduction of proinflammatory cytokines including TNF, which has been shown to inhibit the growth and differentiation of erythroid progenitors in the BM (57–59) and inhibit erythropoietin production (60), and IL-1, which also inhibits erythropoietin production (60) and drives HSCs toward myelopoiesis (61). Of note, the IL-1 receptor signals in part through IL-1 receptor associated kinases (IRAKs) via the adaptor Myd88 also used by plasma membrane TLRs such as TLR-4 (62). Therefore, we first explored the effects of LPS on medullary erythropoiesis in mice lacking IL-1 receptor (*Il1r1*^–^*^/^*mice), or both TNF receptors 1a and 1b (*Tnfrsf1a*^–^*^/^*^–^*; Tnfrsf1b*^–^*^/^*^–^ mice) (Figures 6A,D). The BM was still whitened in LPS-treated *Il1r1*^–^*^/^*^–^ mice and *Tnfrsf1a*^–^*^/^*^–^*; Tnfrsf1b*^–^*^/^*^–^ mice (Figures 6B,E). Likewise, LPS still suppressed medullary erythropoiesis in the absence of IL-1 receptor or TNF-α receptors (Figures 6C,F) suggesting that LPS does not require these inflammatory cytokines to inhibit medullary erythropoiesis.

**FIGURE 7:**
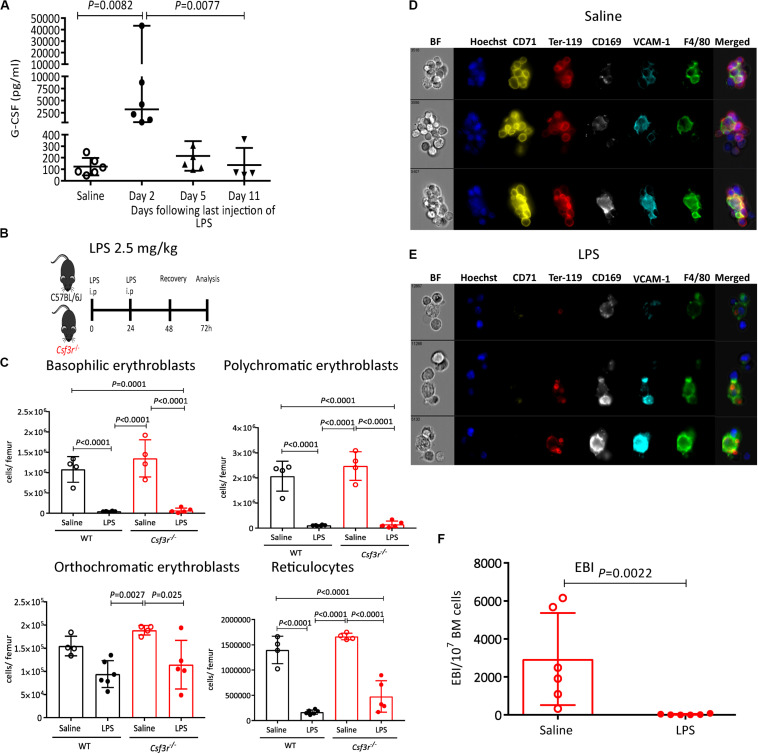
LPS suppresses of medullary erythropoiesis independently of G-CSF receptor. **(A)** G-CSF concentration in blood plasma at various time points following the last LPS injection. **(B)** Wildtype (WT) and GCSF receptor knock out (*Csf3r*^–^*^/^*^–^) mice were administered saline or LPS and harvested after 48 h of recovery. **(C,D)** Representative photomicrographs of gated (Hoechst^+^ CD71^+^ Ter119^+^ F4/80^+^ VCAM1^+^ cell aggregates after saline **(C)** or LPS **(D)** treatment. Note the disruption of typical EBIs after LPS treatment of *Csf3r*^–/–^ mice. **(E)** Quantification of EBI frequency in the BM of *Csf3r*^–/–^mice treated with saline or LPS. **(F)** Quantification by flow cytometry of erythroblasts and reticulocytes in the femoral BM from WT or *Csfr3r*^–^*^/^*^–^ mice. Data are from 2 pooled experiments performed at several month interval. Each dot represents an individual mouse (*n* = 4–6 mice per group) and bars are means ± SD. Statistical significance was determined using Kruskal–Wallis with Dunn’s multiple comparison test in panel a and one-way ANOVA with Tukey’s multiple comparison test in panel **(F)**, and two-tailed Mann–Whitney test in **(E)**.

We have previously reported that administration of G-CSF, a widely used cytokine to mobilize HSCs in the clinic (63), causes similar suppression of medullary erythropoiesis (20) and reduces EBI frequency in the BM (38). It has also been shown that LPS increases splenic hematopoiesis in G-CSF-dependent manner however, whether increased splenic hematopoiesis was mediated through HSC mobilization into the blood or extramedullary hematopoiesis was not investigated (49). To delineate a possible role of G-CSF on LPS-induced medullary anemia, we first measured the effect of LPS on G-CSF release in blood plasma. lipopolysaccharides increased G-CSF plasma concentration, peaking at 25-fold baseline levels two days post LPS treatment (Figure 7A). We next analyzed LPS effect on HSC mobilization and medullary erythropoiesis in *Csf3r*^–/–^ mice defective for G-CSF receptor gene. We first established that LPS HSPCs mobilized into the blood in a G-CSF-dependent manner, as LPS did not mobilize HSPCs in blood or spleen in *Csf3r*^–/–^ mice (Supplementary Figures 8A–F). Surprisingly however, LPS treatment still profoundly suppressed medullary erythropoiesis in *Csf3r*^–/–^ mice similar to WT controls (Figures 7B,C). Likewise, imaging flow cytometry confirmed that LPS sharply decreased EBI frequency in the BM of *Csf3r*^–/–^ mice similar to WT control mice (Figures 7D–F). Therefore, unlike HSPC mobilization, LPS does not require G-CSF signaling to suppress medullary erythropoiesis and is mechanistically distinct from LPS-induced HSPC mobilization.

## Discussion

LPS/zymosan A (15, 16, 43, 47, 64, 65), *Salmonella* (52), *Staphylococcus epidermidis* (66), and *Brucella abortus* (67) have been reported to induce AI in mice. A mechanism frequently invoked is the increase in IL-6 expression during infections or inflammatory challenges that activates hepcidin transcription and secretion by hepatocytes. Hepcidin, a master regulator of iron homeostasis, directly binds to and functionally blocks ferroportin, the iron transporter that allows alimentary iron transport across the intestinal barrier and iron transfer from stores in macrophages to adjacent erythroblasts. From this, it is assumed that AI is in part caused by iron restriction erythropoiesis (68, 69) but in many aspects such as erythrocyte size, hemoglobin load, or iron storage capacity, AI is very different from IDA (11–13). Although earlier studies have reported that LPS reduced the number of nucleated erythroid cells and reticulocytes and iron incorporation in the BM (15, 16), the mechanisms by which LPS suppresses medullary erythropoiesis have not been extensively studied.

We now report that LPS from gram-negative *E. coli* suppresses erythropoiesis in the BM with profound reduction in erythroblasts numbers in a TLR-4- and MyD88-dependent manner but independently of G- CSF-, TNF-, or IL-1-mediated signaling. This effect is rapid, developing within 48 h of LPS challenge. We also give evidence that this effect is indirectly mediated as erythroid progenitors do not express TLR-4 protein or mRNA and LPS does not inhibit BFU-E colony formation *in vitro*. Although our results do not exclude the possible involvement of other cell types expressing TLR-4 and MyD88 to suppress medullary erythropoiesis in response to LPS, our observation that LPS treatment leads to an over 95% reduction of medullary EBI frequency and erythroblasts while proerythroblasts and BFU-E are only reduced by 50%, suggests that medullary erythropoiesis suppression could be largely mediated by EBI Mφ which are at the center of the erythropoietic niche that forms the EBIs. This interpretation is consistent with the observations that Mφ mediate many biological effects of LPS, that their overall morphology is dramatically altered in the BM of LPS-treated mice (Figures 5C,D, 7D,E), and that EBIs are important for erythroblast maturation and enucleation. However future experiments in which the *Tlr4* gene is specifically deleted from Mφ are necessary to definitively confirm this conclusion and precisely delineate the role of macrophages in the acute suppression of medullary EBIs and erythropoiesis in response to LPS challenge.

It is known that inflammation induces stress erythropoiesis in the mouse spleen to maintain erythroid homeostasis (15, 43–47, 70). Likewise, we find that LPS promotes splenic stress erythropoiesis to compensate for reduced medullary erythropoiesis (Supplementary Figure 4). It has recently emerged that mice may be much more prone to compensatory splenic erythropoiesis compared to rats and humans (71). This suggests that LPS may cause a more profound anemia in humans than in mice if splenic compensatory erythropoiesis is not as strongly induced in response to LPS in patients.

It is also important to note that LPS effect in the BM is not limited to erythropoiesis suppression. Previous studies have shown a large expansion of the HSPC pool in the spleen of mice treated with LPS in a G-CSF-dependent manner and inferred that HSPCs were mobilized from the BM into the blood; however they did not examine HSPC mobilization proper into the blood (49), a *sine qua non-condition* for HSPC mobilization leaving the possibility that LPS induced extramedullary hematopoiesis rather than HSPC mobilization. We now definitively demonstrate that HSPCs are actually mobilized into the blood in response to LPS and that this mobilization is indeed TLR-4- and G-CSF-dependent. Therefore, the HSPC mobilization response to LPS, which is G-CSF-dependent, is mechanistically distinct from medullary erythropoiesis suppression which is G-CSF-independent.

In respect to other pro-inflammatory cytokines induced by LPS treatment such as TNF, IL-1β, and G-CSF, TNF inhibits HSC proliferation and repopulating capacity *in vivo* (72), inhibits differentiation of BM erythroid progenitors, shortens mature erythrocyte lifespan (58, 59, 73) and inhibits erythropoietin production (60) whereas IL-1β, which is released as a mature peptide following NLRP3 inflammasome activation and pyroptosis of Mφ (74), forces HSC myeloid differentiation at the expense of self-renewal (61) and reduces erythroid colony-formation *in vitro* (75) via interferon-γ (76). Interestingly IL-18, which is also produced as an active peptide following LPS priming and NLRP3 inflammation activation (74), also induces interferon-γ expression (77) similar to IL-1β. However a possible role of IL-18 in regulating erythropoiesis remains to our knowledge unknown and its possible role in erythropoiesis suppression by LPS remains to be determined. IL-1α, IL-1β, and TNF have all been reported to inhibit erythropoietin production by the Hep3B cell line (60). Despite these and the fact that Mφ secrete TNF and IL-1β upon stimulation by LPS (and additional inflammasome activation for IL-1β), we find that LPS-mediated suppression of medullary erythropoiesis was not dependent on either TNF-α or IL-1 receptor signaling. There are therefore other mechanisms at work to suppress medullary erythropoiesis in response to LPS.

In Mφ, TLR-4 activates two distinct signaling cascades mediated by two adaptor proteins carrying TIR (Toll-interleukin receptor) domains, namely MyD88 and TRIF (toll-like receptor adaptor molecule 1) depending of the subcellular location of TLR-4 (78). lipopolysaccharide activates both MyD88-dependent (79, 80) and MyD88-independent pathways mediated via TRIF (81, 82). MyD88 is essential for LPS-induced emergency granulopoiesis (83), myelosuppression (84) and *Salmonella-*induced stress erythropoiesis (52). Likewise, we have found that LPS required MyD88 for the inhibition of medullary erythropoiesis (Figure 2). In contrast, LPS impairs HSCs repopulation ability and causes proliferative stress via TRIF but not MyD88 (50). TRIF signaling is also crucial for LPS-induced HSC injury and impairing HSC functions (84). Therefore these findings together with ours suggest that the effects of LPS on HSCs and medullary erythropoiesis are mediated by distinct signaling pathways.

In conclusion, we have found that systemic LPS profoundly reduces EBI frequency and erythropoiesis in the BM in an erythroid cell extrinsic, TLR-4- and MyD88-dependent manner, but independently of G-CSF, IL-1, and TNF signaling. Our work suggests that EBI Mφ in the BM may be responsible for this effect by sensing innate immune stimuli in response to acute inflammation and infections to rapidly convert to pro-inflammatory function at the expense of their pro-erythropoietic function. This conclusion will need to be confirmed in mice in which the *Tlr4* gene is specifically depleted in Mφ rather than the germinal deletion used in our study.

## Data Availability Statement

The raw data supporting the conclusions of this article will be made available by the authors, without undue reservation.

## Ethics Statement

The animal study was reviewed and approved by Animal Experimentation Committee of the University of Queensland.

## Author Contributions

KB coordinated the work, planned and performed the experiments, analyzed the data, interpreted results, and wrote and edited the manuscript. JT performed imaging flow cytometry experiments. CM performed the animal experiments, BFU-E colony assays and edited the manuscript. WF assisted with animal experiments and qRT-PCR assays. RW, VB, and BN performed some of the experiments. IW edited the manuscript. J-PL conceived the work, helped with flow cytometry design and analyses, interpreted the results, and wrote and edited the manuscript. All authors contributed to the article and approved the submitted version.

## Conflict of Interest

The authors declare that the research was conducted in the absence of any commercial or financial relationships that could be construed as a potential conflict of interest.

## Footnotes

1 https://gexc.riken.jp

2 https://haemosphere.org
