# Supplementary material for: Bacterial Lipopolysaccharides Suppress Erythroblastic Islands and Erythropoiesis in the Bone Marrow in an Extrinsic and G- CSF-, IL-1-, and TNF-Independent Manner
Source: Front Immunol. 2020 Oct 6;11:583550. doi: 10.3389/fimmu.2020.583550 (PMC7573160; doi:10.3389/fimmu.2020.583550)
Supplement: Supplementary file 1 [file Data_Sheet_1.PDF]

Supplementary Table 1. Fluorescent antibodies used for flow cytometry.

**HSPC stain**	**Antibody**	**Cat#**	**Lot#**	**Company**	**Clone**	**Dilutions**
	CD3ε-FITC	100306	B215536	BioLegend	145-2C11	(1/300)
	CD5-FITC	100606	B159705	BioLegend	53-7.3	(1/300)
	B220 (CD45R)-FTIC	103206	B230445	BioLegend	RA3-6B2	(1/300)
	CD11b-FITC	101206	B224362	BioLegend	M1/70	(1/300)
	Gr1-FITC	108406	B193722	BioLegend	RB6-8C5	(1/300)
	Ter119-FITC	116206	B246091	BioLegend	Ter119	(1/300)
	KIT (CD117)-APC	105812	B199149	BioLegend	2B8	(1/300)
	SCA1-PECY7	108114	B260282	BioLegend	D7	(1/300)
	CD48-PacBlue	103418	B218789	BioLegend	HM48-1	(1/200)
	CD150-PE	115904	B210719	BioLegend	TC15-12F 12.2	(1/200)
	CD45-APCCY7	103116	B257634	BioLegend	30-F11	(1/200)
	FLT3-PECF594	BD 562537	7299980	BD Bioscience	A2F10	(1/150)
**Erythropoiesis stain**	**Antibody**	**Cat#**	**Lot#**	**Company**	**Clone**	**Dilutions**
	Ter119-FITC	116206	B246091	BioLegend	Ter119	(1/200)
	CD44-APC	103012	B186443	BioLegend	IM7	(1/300)
	CD45-APCCY7	103116	B257634	BD Bioscience	30-F11	(1/200)
	Hoechst 33342	62249		ThermoFisher Scientific		1/400
**Macrophage stain**	**Antibody**	**Cat#**	**Lot#**	**Company**	**Clone**	**Dilutions**
	Ter119- PerCPCy5.5	116228	B257070	BioLegend	Ter119	(1/150)
	CD11b-BV510	101245	B253262	BioLegend	M1/70	(1/200)
	F4/80-APC	123116	B264228	BioLegend	BM8	(1/200)

	LY6G-FITC	127606	B277116	BioLegend	1A8	(1/200)
	VCAM-1-PECY7	105720	B190413	BioLegend	429 (MVCAM.A)	(1/150)
	CD169-PE	142404	B178247	BioLegend	3D6.112	1/100
	LY6C-PacBlue	128014	B256779	BioLegend	HK1.4	(1/300)
	CD45-BV785	103149	B278525	BD Bioscience	30-F11	(1/200)
**Erythroid progenitor stain**	**Antibody**	**Cat#**	**Lot#**	**Company**	**Clone**	**Dilutions**
	CD3ε-Biotin	100304	B181424	BioLegend	145-2C11	(1/300)
	CD5-Biotin	100604	B151873	BioLegend	53-7.3	(1/300)
	B220 (CD45R)-Biotin	103204	B164399	BioLegend	RA3-6B2	(1/300)
	CD11b-Biotin	101204	B153498	BioLegend	M1/70	(1/300)
	Gr1-Biotin	108404	B163298	BioLegend	RB6-8C5	(1/300)
	Ter119-Biotin	116204	B168171	BioLegend	Ter119	(1/300)
	KIT(CD117)-APCCy7	105826	B231077	BioLegend	2B8	(1/300)
	SCA1-PECY5	108109	B253664	BioLegend	D7	(1/300)
	CD48-PacBlue	103418	B218789	BioLegend	HM48-1	1/150
	CD150-PECy7	115914	B119751	BioLegend	TC15-12F 12.2	(1/200)
	CD41-FITC	133903	B201956	BioLegend	MWReg30	(1/300)
	CD105-APC	120413	B171413	BioLegend	MJ7/18	(1/200)
	CD16/32- PerCPCy5.5	101324	B250025	BioLegend	93	(1/100)
	CD45-BV785	103149	7096544	BioLegend	30-F11	(1/200)
	Streptavidin-BUV395	564176	7096544	BioLegend		(1/300)
**EBI Mac stain**	**Antibody**	**Cat#**	**Lot#**	**Company**	**Clone**	**Dilutions**
	Ter119- PerCPCy5.5	116228	B257070	BioLegend	Ter119	Jan-40
	CD11b-PECF594	562287	6277958	BioLegend	M1/70	1/100
	F4/80-APC	123116	B264228	BioLegend	BM8	1/150
	Ly6G-APCCy7	127624	B238265	BioLegend	1A8	1/100

VCAM-1-PECY7	105720	B190413	BioLegend	429 (MVCAM.A)	(1/10)
CD169-FITC	142406	B185155	BioLegend	3D6.112	(1/50)
CD71-PE	113807	B178341	BioLegend	R17217	(1/100)
TLR-4-PE	117605	B144258	BioLegend	MTS510	(1/75)
FVS700	564997		BD Bioscience		(1/10000)

Antibody	Cat#	Lot#	Company	Clone	Dilutions
TLR-4-PE	117605	B144258	BioLegend	MTS510	(1/75)
FVS700	564997		BD Bioscience		(1/10000)
7-AAD)	A1310		ThermoFisher Scientific		(1/10)
