# Supplementary material for: Bacterial Lipopolysaccharides Suppress Erythroblastic Islands and Erythropoiesis in the Bone Marrow in an Extrinsic and G- CSF-, IL-1-, and TNF-Independent Manner
Source: Front Immunol. 2020 Oct 6;11:583550. doi: 10.3389/fimmu.2020.583550 (PMC7573160; doi:10.3389/fimmu.2020.583550)
Supplement: Supplementary file 2 [file Presentation_1.pptx]

## Slide 1
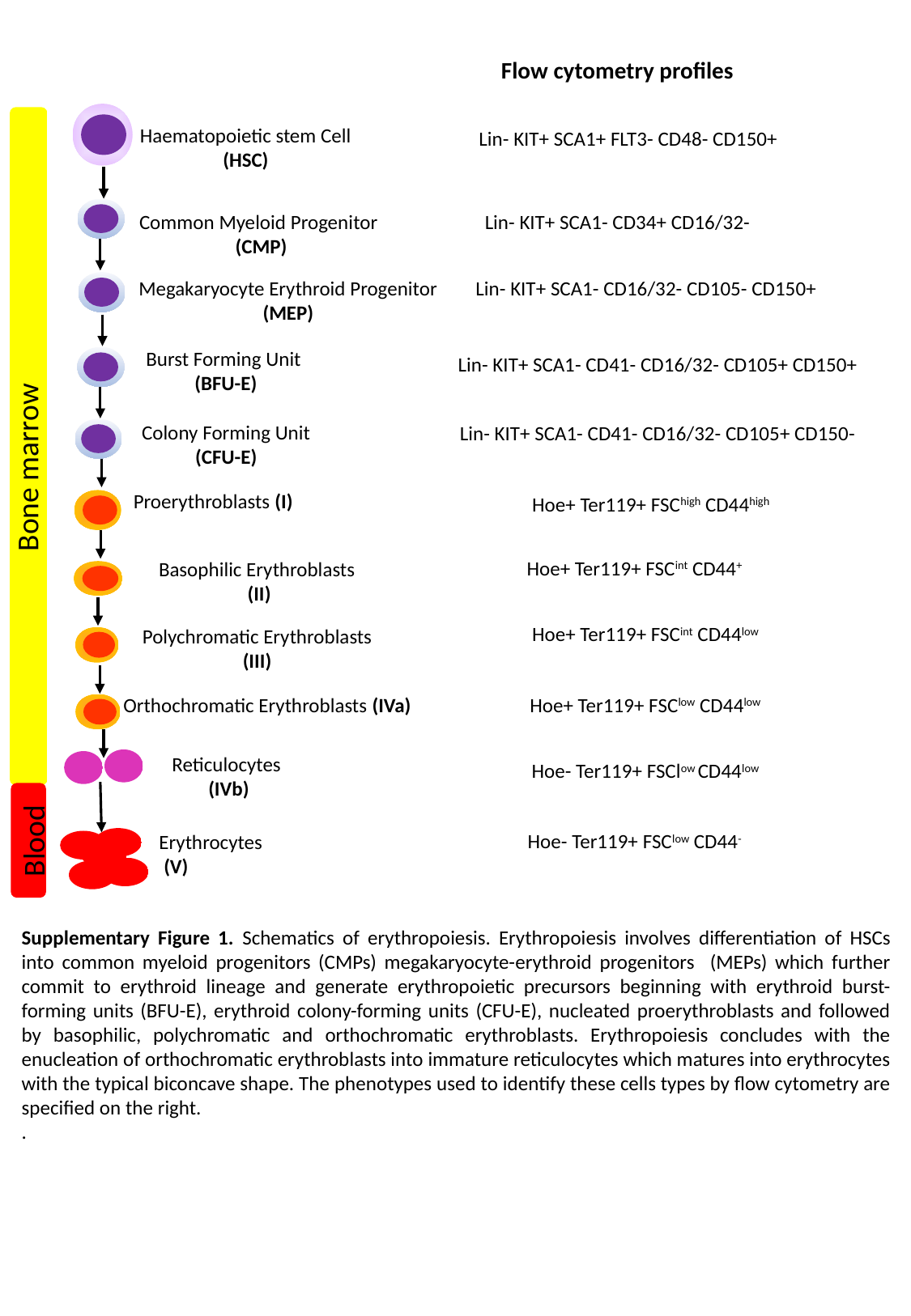

Flow cytometry profiles
Bone marrow
Blood
Haematopoietic stem Cell (HSC)
Lin- KIT+ SCA1+ FLT3- CD48- CD150+
Common Myeloid Progenitor
(CMP)
Lin- KIT+ SCA1- CD34+ CD16/32-
Megakaryocyte Erythroid Progenitor (MEP)
Lin- KIT+ SCA1- CD16/32- CD105- CD150+
Burst Forming Unit
(BFU-E)
Lin- KIT+ SCA1- CD41- CD16/32- CD105+ CD150+
Colony Forming Unit (CFU-E)
Lin- KIT+ SCA1- CD41- CD16/32- CD105+ CD150-
Proerythroblasts (I)
Hoe+ Ter119+ FSChigh CD44high
Hoe+ Ter119+ FSCint CD44+
Basophilic Erythroblasts
(II)
Hoe+ Ter119+ FSCint CD44low
Polychromatic Erythroblasts
(III)
Hoe+ Ter119+ FSClow CD44low
Orthochromatic Erythroblasts (IVa)
Reticulocytes
(IVb)
Hoe- Ter119+ FSClow CD44low
Hoe- Ter119+ FSClow CD44-
Erythrocytes
 (V)
Supplementary Figure 1. Schematics of erythropoiesis. Erythropoiesis involves differentiation of HSCs into common myeloid progenitors (CMPs) megakaryocyte-erythroid progenitors (MEPs) which further commit to erythroid lineage and generate erythropoietic precursors beginning with erythroid burst-forming units (BFU-E), erythroid colony-forming units (CFU-E), nucleated proerythroblasts and followed by basophilic, polychromatic and orthochromatic erythroblasts. Erythropoiesis concludes with the enucleation of orthochromatic erythroblasts into immature reticulocytes which matures into erythrocytes with the typical biconcave shape. The phenotypes used to identify these cells types by flow cytometry are specified on the right.
.

## Slide 2
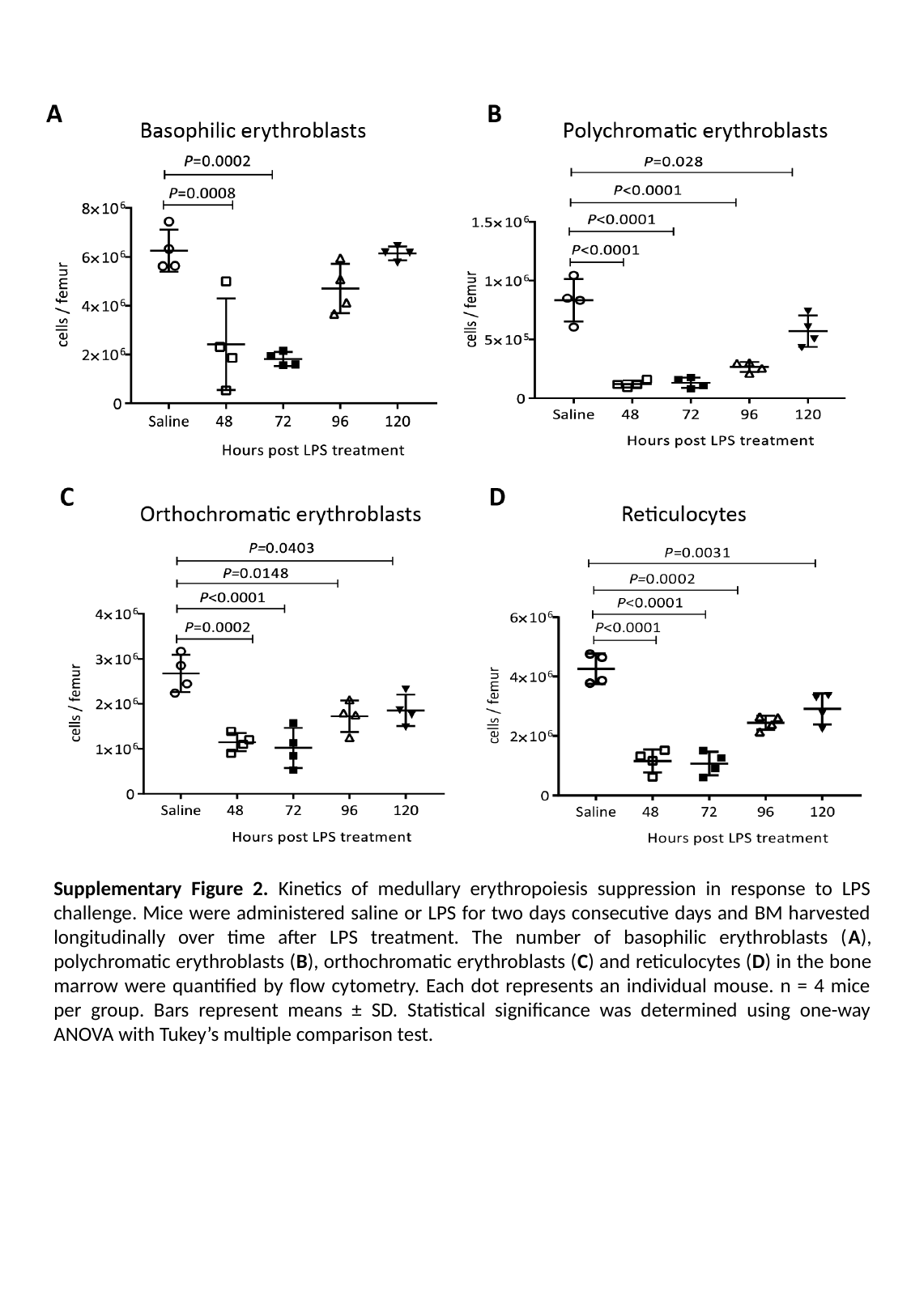

Supplementary Figure 2. Kinetics of medullary erythropoiesis suppression in response to LPS challenge. Mice were administered saline or LPS for two days consecutive days and BM harvested longitudinally over time after LPS treatment. The number of basophilic erythroblasts (A), polychromatic erythroblasts (B), orthochromatic erythroblasts (C) and reticulocytes (D) in the bone marrow were quantified by flow cytometry. Each dot represents an individual mouse. n = 4 mice per group. Bars represent means ± SD. Statistical significance was determined using one-way ANOVA with Tukey’s multiple comparison test.

## Slide 3
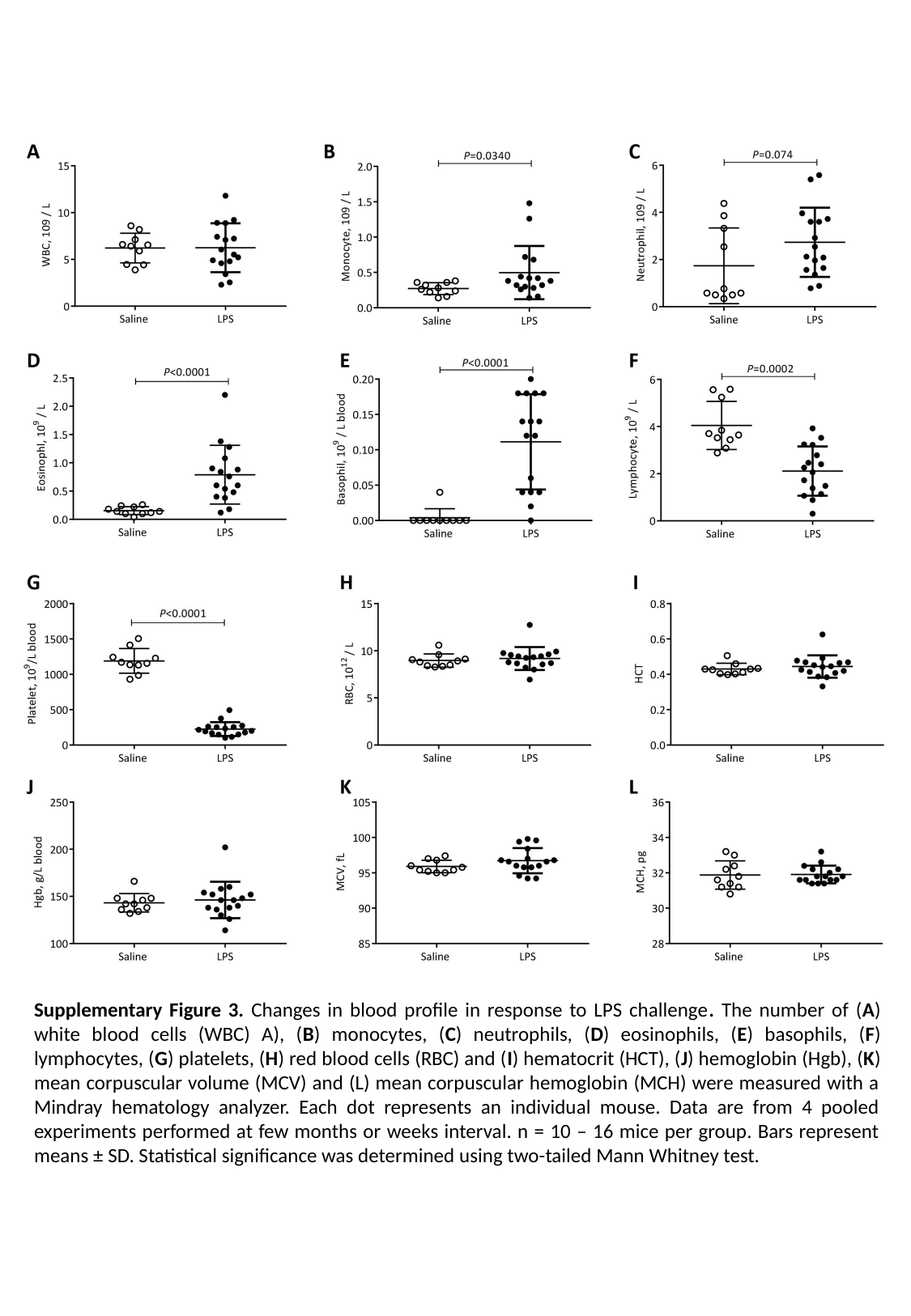

Supplementary Figure 3. Changes in blood profile in response to LPS challenge. The number of (A) white blood cells (WBC) A), (B) monocytes, (C) neutrophils, (D) eosinophils, (E) basophils, (F) lymphocytes, (G) platelets, (H) red blood cells (RBC) and (I) hematocrit (HCT), (J) hemoglobin (Hgb), (K) mean corpuscular volume (MCV) and (L) mean corpuscular hemoglobin (MCH) were measured with a Mindray hematology analyzer. Each dot represents an individual mouse. Data are from 4 pooled experiments performed at few months or weeks interval. n = 10 – 16 mice per group. Bars represent means ± SD. Statistical significance was determined using two-tailed Mann Whitney test.

## Slide 4
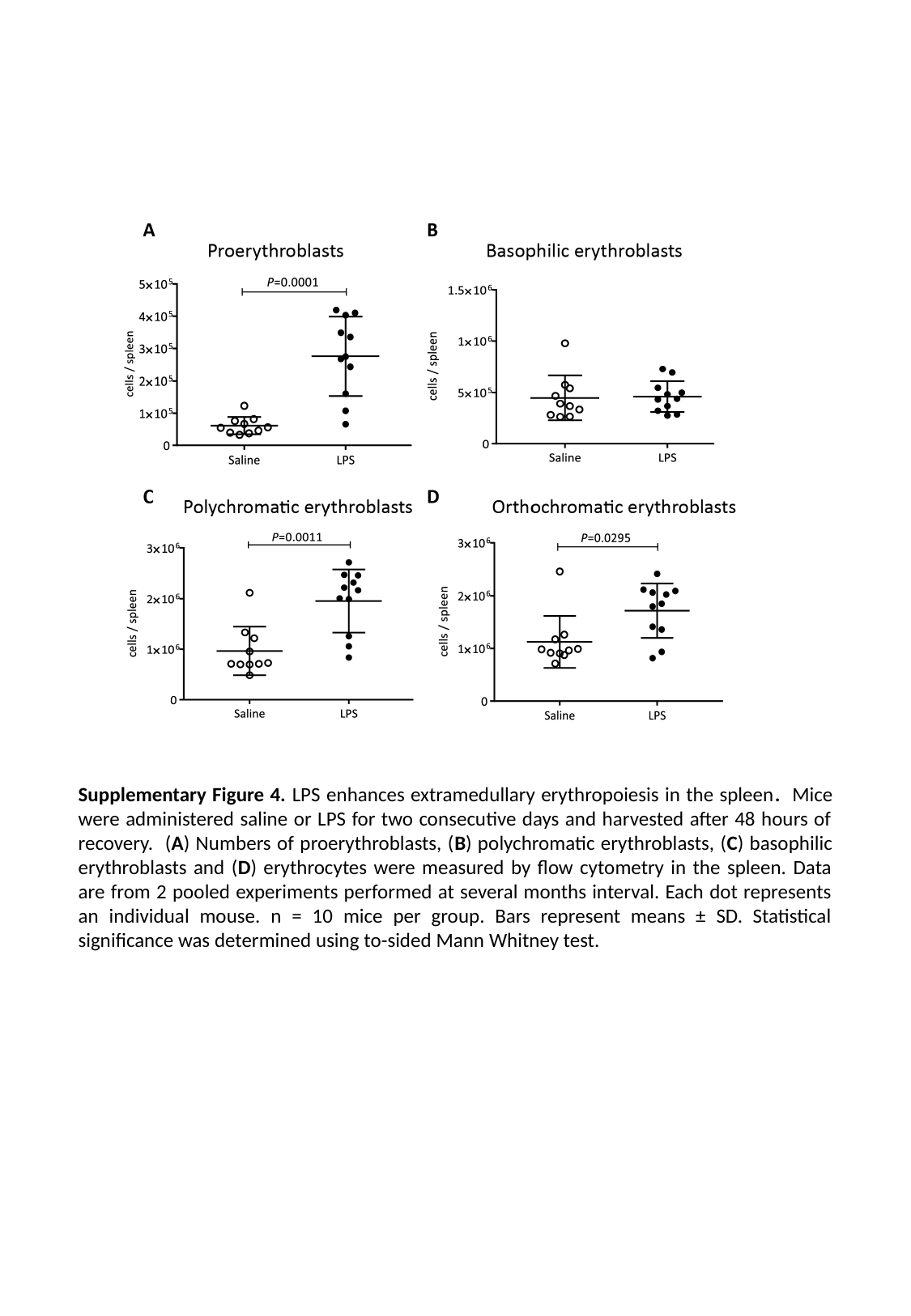

Supplementary Figure 4. LPS enhances extramedullary erythropoiesis in the spleen. Mice were administered saline or LPS for two consecutive days and harvested after 48 hours of recovery. (A) Numbers of proerythroblasts, (B) polychromatic erythroblasts, (C) basophilic erythroblasts and (D) erythrocytes were measured by flow cytometry in the spleen. Data are from 2 pooled experiments performed at several months interval. Each dot represents an individual mouse. n = 10 mice per group. Bars represent means ± SD. Statistical significance was determined using to-sided Mann Whitney test.

## Slide 5
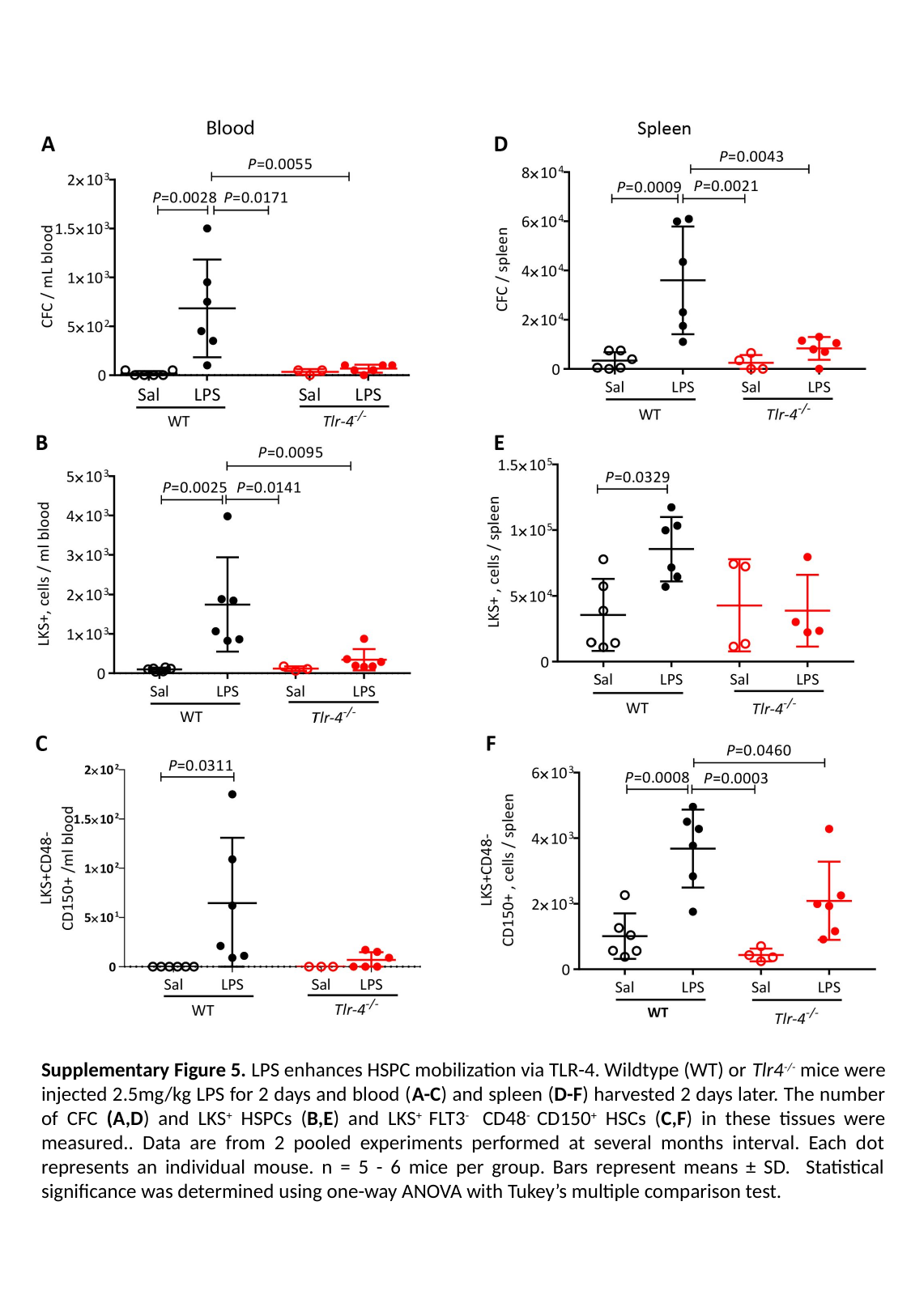

Supplementary Figure 5. LPS enhances HSPC mobilization via TLR-4. Wildtype (WT) or Tlr4-/- mice were injected 2.5mg/kg LPS for 2 days and blood (A-C) and spleen (D-F) harvested 2 days later. The number of CFC (A,D) and LKS+ HSPCs (B,E) and LKS+ FLT3- CD48- CD150+ HSCs (C,F) in these tissues were measured.. Data are from 2 pooled experiments performed at several months interval. Each dot represents an individual mouse. n = 5 - 6 mice per group. Bars represent means ± SD. Statistical significance was determined using one-way ANOVA with Tukey’s multiple comparison test.

## Slide 6
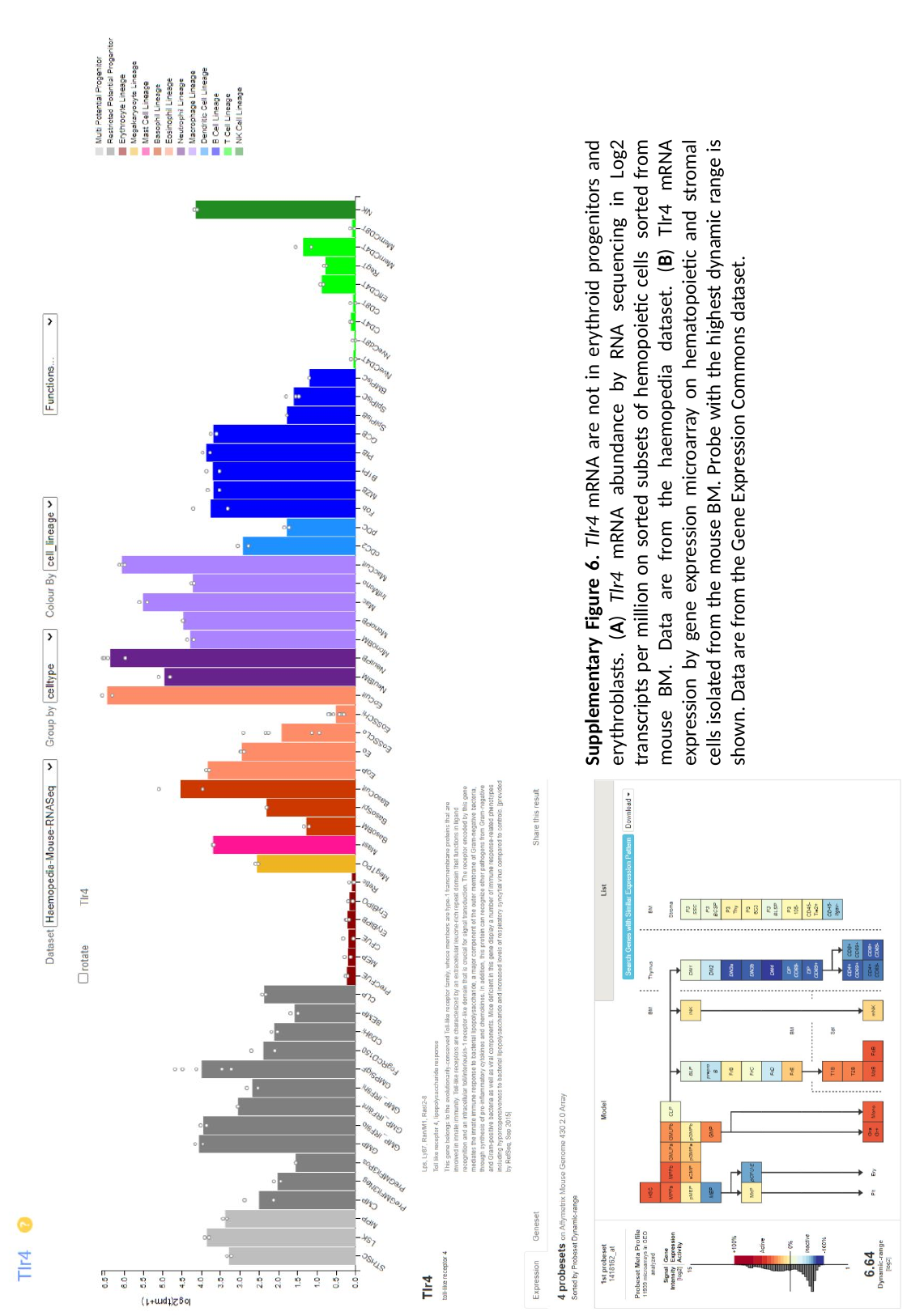

Supplementary Figure 6. Tlr4 mRNA are not in erythroid progenitors and erythroblasts. (A) Tlr4 mRNA abundance by RNA sequencing in Log2 transcripts per million on sorted subsets of hemopoietic cells sorted from mouse BM. Data are from the haemopedia dataset. (B) Tlr4 mRNA expression by gene expression microarray on hematopoietic and stromal cells isolated from the mouse BM. Probe with the highest dynamic range is shown. Data are from the Gene Expression Commons dataset.

## Slide 7
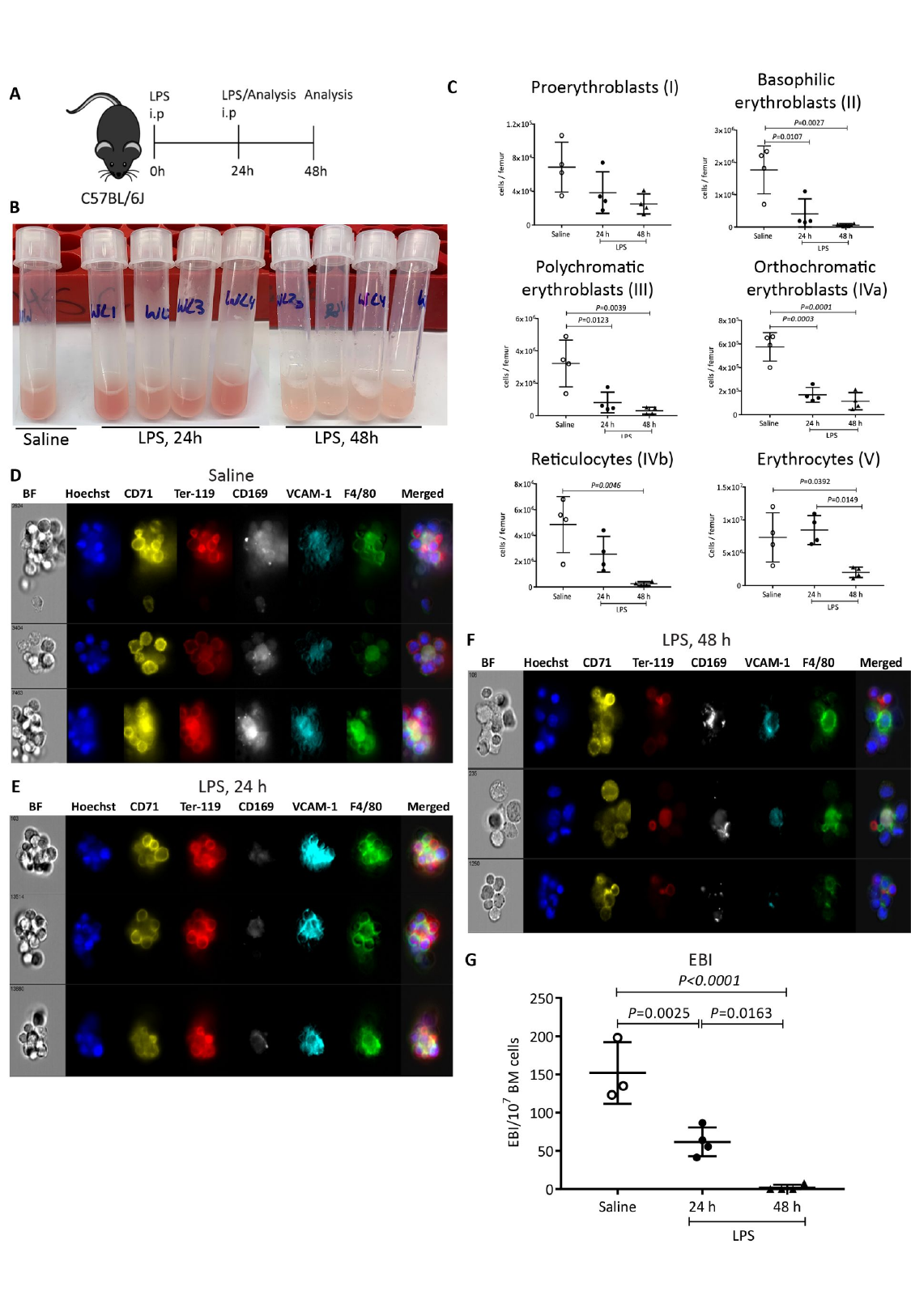

## Slide 8
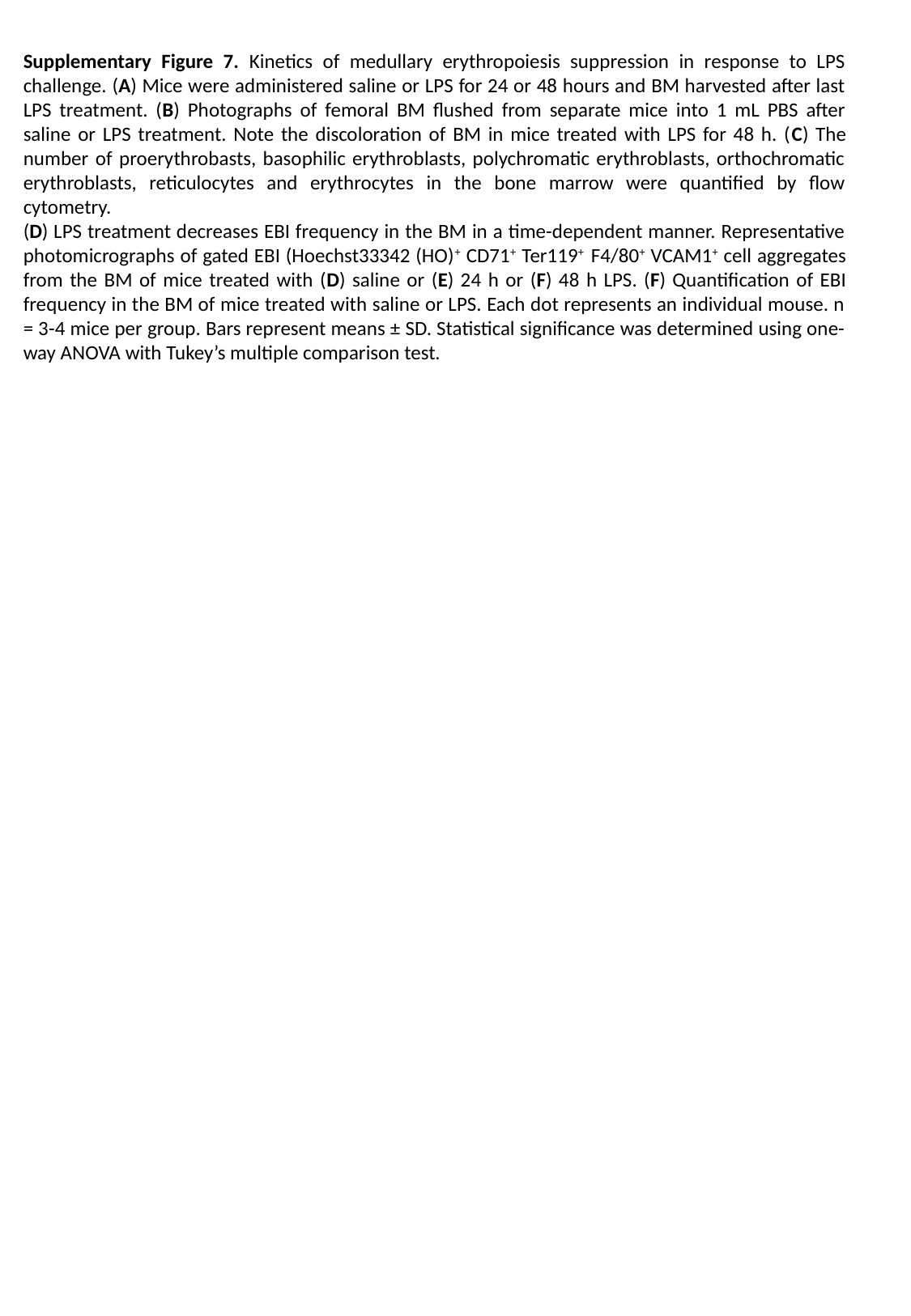

Supplementary Figure 7. Kinetics of medullary erythropoiesis suppression in response to LPS challenge. (A) Mice were administered saline or LPS for 24 or 48 hours and BM harvested after last LPS treatment. (B) Photographs of femoral BM flushed from separate mice into 1 mL PBS after saline or LPS treatment. Note the discoloration of BM in mice treated with LPS for 48 h. (C) The number of proerythrobasts, basophilic erythroblasts, polychromatic erythroblasts, orthochromatic erythroblasts, reticulocytes and erythrocytes in the bone marrow were quantified by flow cytometry.
(D) LPS treatment decreases EBI frequency in the BM in a time-dependent manner. Representative photomicrographs of gated EBI (Hoechst33342 (HO)+ CD71+ Ter119+ F4/80+ VCAM1+ cell aggregates from the BM of mice treated with (D) saline or (E) 24 h or (F) 48 h LPS. (F) Quantification of EBI frequency in the BM of mice treated with saline or LPS. Each dot represents an individual mouse. n = 3-4 mice per group. Bars represent means ± SD. Statistical significance was determined using one-way ANOVA with Tukey’s multiple comparison test.

## Slide 9
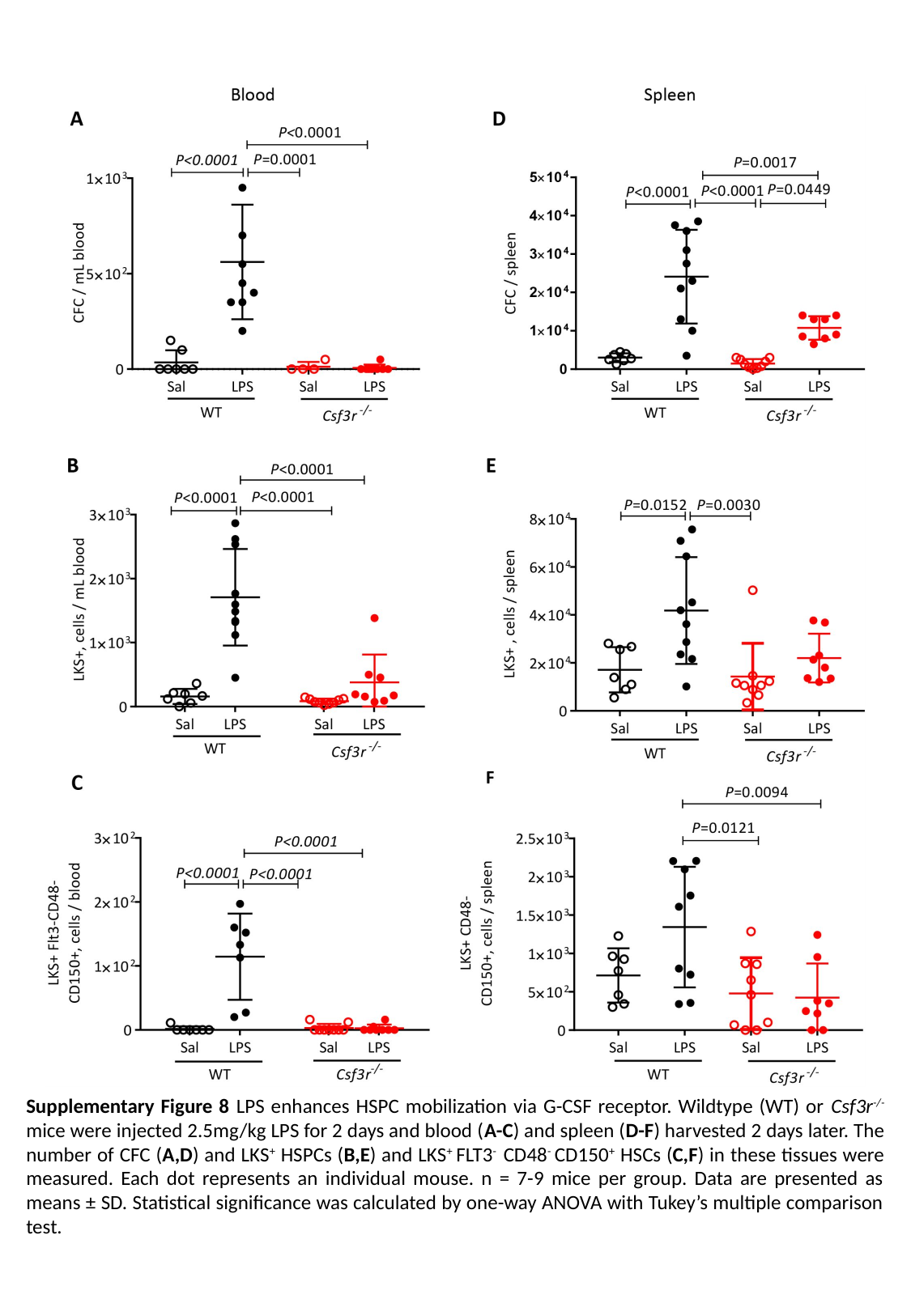

Supplementary Figure 8 LPS enhances HSPC mobilization via G-CSF receptor. Wildtype (WT) or Csf3r-/- mice were injected 2.5mg/kg LPS for 2 days and blood (A-C) and spleen (D-F) harvested 2 days later. The number of CFC (A,D) and LKS+ HSPCs (B,E) and LKS+ FLT3- CD48- CD150+ HSCs (C,F) in these tissues were measured. Each dot represents an individual mouse. n = 7-9 mice per group. Data are presented as means ± SD. Statistical significance was calculated by one-way ANOVA with Tukey’s multiple comparison test.
